# Post-translational Serine/Threonine Phosphorylation and Lysine Acetylation: A Novel Regulatory Aspect of the Global Nitrogen Response Regulator GlnR in *S. coelicolor* M145

**DOI:** 10.3389/fmolb.2016.00038

**Published:** 2016-08-09

**Authors:** Rafat Amin, Mirita Franz-Wachtel, Yvonne Tiffert, Martin Heberer, Mohamed Meky, Yousra Ahmed, Arne Matthews, Sergii Krysenko, Marco Jakobi, Markus Hinder, Jane Moore, Nicole Okoniewski, Boris Maček, Wolfgang Wohlleben, Agnieszka Bera

**Affiliations:** ^1^Department of Pathology, Dow International Medical College, Dow Research Institute of Biotechnology and Biomedical Sciences, Dow University of Health SciencesKarachi, Pakistan; ^2^Proteome Center Tübingen, Interdepartmental Institute for Cell Biology (IFIZ), University of TübingenTübingen, Germany; ^3^B.R.A.I.N. Biotechnology Research and Information Network AGZwingenberg, Germany; ^4^Microbiology and Biotechnology, Interfaculty Institute of Microbiology and Infection Medicine, University of TübingenTübingen, Germany; ^5^Department of Pharmaceutical Biotechnology, Helmholtz Institute for Pharmaceutical Research Saarland, Saarland University CampusSaarbrücken, Germany; ^6^John Innes Center, Norwich Research ParkNorwich, UK

**Keywords:** nitrogen assimilation, *Streptomyces coelicolor*, GlnR, regulation, post-translational modifications, acetylation, phosphorylation

## Abstract

Soil-dwelling *Streptomyces* bacteria such as *S*.coelicolor have to constantly adapt to the nitrogen (*N*) availability in their habitat. Thus, strict transcriptional and post-translational control of the *N*-assimilation is fundamental for survival of this species. GlnR is a global response regulator that controls transcription of the genes related to the *N*-assimilation in *S. coelicolor* and other members of the *Actinomycetales*. GlnR represents an atypical orphan response regulator that is not activated by the phosphorylation of the conserved aspartate residue (Asp 50). We have applied transcriptional analysis, LC-MS/MS analysis and electrophoretic mobility shift assays (EMSAs) to understand the regulation of GlnR in *S. coelicolor* M145. The expression of *glnR* and GlnR-target genes was revisited under four different *N*-defined conditions and a complex *N*-rich condition. Although, the expression of selected GlnR-target genes was strongly responsive to changing *N-concentrations*, the *glnR* expression itself was independent of the *N*-availability. Using LC-MS/MSanalysis we demonstrated that GlnR was post-translationally modified. The post-translational modifications of GlnR comprise phosphorylation of the serine/threonine residues and acetylation of lysine residues. In the complex *N*-rich medium GlnR was phosphorylated on six serine/threonine residues and acetylated on one lysine residue. Under defined *N*-excess conditions only two phosphorylated residues were detected whereas under defined *N*-limiting conditions no phosphorylation was observed. GlnR phosphorylation is thus clearly correlated with *N*-rich conditions. Furthermore, GlnR was acetylated on four lysine residues independently of the *N*-concentration in the defined media and on only one lysine residue in the complex *N*-rich medium. Using EMSAs we demonstrated that phosphorylation inhibited the binding of GlnR to its targets genes, whereas acetylation had little influence on the formation of GlnR-DNA complex. This study clearly demonstrated that GlnR DNA-binding affinity is modulated by post-translational modifications in response to changing *N*-conditions in order to elicit a proper transcriptional response to the latter.

## Introduction

Streptomycetes, like other microorganisms, need to accurately modulate their regulatory network according to their developmental stage while simultaneously responding to environmental changes, such as the continuous variation in nutrient availability, including nitrogen (*N*), in the soil habitat. Therefore, strict transcriptional and post-translational control of the *N*-assimilation is fundamental for survival of streptomycetes. Transcriptional regulation of the *N*-assimilation in streptomycetes is accomplished by GlnR (global nitrogen response regulator; Tiffert et al., 2008; Pullan et al., 2011). However, control of the *N*-metabolism by GlnR is not only restricted to streptomycetes, since conserved GlnR homologs were also found in other actinomycetes such as: *Mycobacterium* sp., *Amycolatopsis* sp., *Saccharopolyspora* sp., *Bifidobacterium* sp., *Frankia* sp., *Nocardia* sp., *Propionibacterium* sp., and *Rhodococcus* sp. (Amon et al., 2009), signifying evolutionary importance of this regulator. The GlnR regulator in *S. coelicolor* controls at least 10 genes which are directly involved in *N*-metabolism and seven additional genes encoding proteins of unknown function (Reuther and Wohlleben, 2007; Tiffert et al., 2008; Wang and Zhao, 2009; Amin et al., 2012). Proteomic analysis demonstrated a more comprehensive regulatory role of GlnR in connection with central carbon metabolic pathways in *S. coelicolor* M145 (Tiffert et al., 2011). Over 50 proteins associated with amino acid biosynthesis and carbon metabolism were shown to be differentially expressed between *S. coelicolor* M145 and the *glnR* mutant (Tiffert et al., 2011). GlnR-mediated control of carbohydrate transport (Liao et al., 2015) and regulation of ectoin (Shao et al., 2015) as well as validomycin A production (Qu et al., 2015) extended the GlnR role beyond the regulation of the *N*-assimilation. Regulation of the *N*-metabolism in *S. coelicolor* is very complex and depending on the conditions, involves additional control by other regulators such as: GlnRII (Fink et al., 2002; Reuther and Wohlleben, 2007), PhoP (Rodríguez-García et al., 2009; Martín et al., 2011; Sola-Landa et al., 2013), Crp (Gao et al., 2012), and AfsQ1 (Wang R. et al., 2013).

Although, the expression of the GlnR target genes in *S. coelicolor* (Tiffert et al., 2008) and other actinomycetes was extensively studied (Pullan et al., 2011; Jenkins et al., 2013; Yao et al., 2014; Williams et al., 2015), little is known on how GlnR controls expression of its target genes according to changing *N*-conditions and thus how the DNA-binding activity of GlnR is modulated. The GlnR regulator belongs to the OmpR-family of transcriptional response regulators, commonly existing as a two component systems with a cognate histidine kinase. Usually the histidine kinase autophosphorylates upon reception of an unknown signal from the environment and subsequently phosphorylates a conserved aspartic acid residue in the receiver domain, of the cognate response regulator, generating an appropriate adaptive response. The typical “phosphorylation pocket” of the response regulator OmpR is composed of six essential residues: the phosphor-accepting aspartate (Asp 55), three catalytic residues (Asp 11, Asp12, and Lys 105) and two conformational switch residues (Thr 87 and Tyr 106; Brissette et al., 1991). GlnR possess only two out of six conserved residues, namely Asp 50 and Thr 79 equivalent to Asp 55 and Thr 87 in OmpR, respectively. The analysis of the partial crystal structures of GlnR from *A. mediterranei* and *M. smegmatis* as well as the structure-based sequence alignment of GlnR from *S. coelicolor*, demonstrated that GlnR not only lacks the typical “phosphorylation pocket” but it is also not phosphorylated at the conserved Asp 50 residue (Lin et al., 2014). However, the conserved Asp50 residue is critical for GlnR homodimerization via its charge interactions with the surrounding residues and is essential for the physiological function of GlnR as shown by *in vitro* and *in vivo* studies (Lin et al., 2014). Furthermore, GlnR is an “orphan” response regulator since no associated sensor kinase gene could be found in its close proximity in the *S. coelicolor* M145 genome. So, since GlnR is not activated by the classical phosphorylation observed for canonical OmpR/PhoP—family members, important question remains still unanswered: how this regulator is activated? How does *S. coelicolor* sense the availability of different *N*-sources sources and how does GlnR elicit the proper transcriptional response according to changing *N*-conditions in the environment? In this study we report for the first time the phosphorylation of the global nitrogen regulator GlnR on serine/threonine residues as well as its acetylation on lysine residues. We also demonstrated that such unusual post-translational modifications play a crucial role in the regulation of the GlnR DNA-binding activity.

## Materials and methods

### Bacterial strains, plasmids, and growth conditions

Strains and plasmids used in this study are listed in Table 1. *E. coli* strains were cultivated either on a solid or in a liquid Luria-Bertani (LB) medium at 37°C (Sambrook et al., 1989). *Streptomyces coelicolor* M145 was cultivated at 30°C on R2YE agar or Mannitol Soy flour (MS) agar (Kieser et al., 2000). For growth in liquid medium, complex S-medium (Okanishi et al., 1974), and defined Evans medium (Evans et al., 1970) was used. Carbon to nitrogen ratio was set as follows: for *N*-limitation in Evans medium C:N of 2 and for *N*-excess in Evans medium C:N of 60. Media was supplemented when appropriate with: ampicillin (150 μg/ml), kanamycin (50 μg/ml), chloramphenicol (25 μg/ml), nalidixic acid (255 μg/ml) or thiostrepton (12.5 μg/ml), unless otherwise stated. Genetic manipulation of *S. coelicolor* M145 and *E. coli* was performed as described by (Kieser et al., 2000) and (Sambrook et al., 1989), respectively.

**Table 1:** **Strains and plasmids used in this study**.

**Strains**	**Genotype**	**References**
*E. coli* BL21 (DE3)	F^−^, *dcm, ompT, hsdS*(r*_B_*− m*_B−_*), *gal*, (DE3)	Studier and Moffatt, 1986
*E. coli* BL21 pET15b-His-CobB1	His-CobB1 overexpression strain Cm^R^, Amp^R^	This work
*E. coli* BL21 pET15b-His-CobB2	His-CobB2 overexpression strain Cm^R^, Amp^R^	This work
*S. coelicolor* M145	*S. coelicolor A3(2) spc1^−^* and *spc2^−^*	Kieser et al., 2000
*S. coelicolor* M145 *glnR*	*glnR* mutant strain of *S. coelicolor M145; glnR* replaced by an *aac(3)IV* cassette, Apr^R^	Tiffert et al., 2011
*S. coelicolor* M145 pGMStrep-*glnR*	*S. coelicolor* M154 with pGM-Strep*-glnR*, P_*tipA*_, Km^R^	Tiffert et al., 2008
pGM190	*Streptomyces- E.coli* shuttle vector, Ts^R^, Km^R^, pSG5 derivative, P_*tipA*_	Wohlleben et al., 2009
pET15b	His-(*N*-term), Amp^R^, P_*tipA*_	Novagen

### RT-PCR

For the transcriptional analysis experiments, the *S. coelicolor* M145 wild type and the *glnR* mutant were grown in the complex S-medium for 4 days at 30°C. After 4 days, cells were harvested and washed twice with the defined Evans medium without *N*-source to remove traces of the S-medium. The biomass was subsequently transferred into the defined Evans medium supplemented with variable concentrations of either ammonium (low: 5 mM, high: 100 mM) or nitrate (low: 5 mM, high: 100 mM) and glucose (low: 2.5 g/l or high: 25 g/l). RT-PCR was conducted using total RNA isolated from *S. coelicolor* M145 and the *glnR* mutant after 24 h of growth in defined Evans medium. The RNA isolation was performed with an RNeasy kit (Qiagen). All RNA preparations were treated twice with DNase (Fermentas). First, an on-column digestion was carried out for 30 min at 24°C, and afterwards RNA samples were treated with DNase for 1.5 h at 37°C. RNA concentrations and quality were checked using a NanoDrop ND-1000 spectrophotometer (Thermo Fisher Scientific). The cDNA from 3 μg RNA was generated with random nonamer primers (Sigma), reverse transcriptase and cofactors (Fermentas). The reverse transcription products (1 μl) were then used as template for PCR amplification. A standard PCR protocol using Taq DNA polymerase (GENAXXON bioscience) and primers annealing to internal parts of the various genes was used. Primers targeting *hrdB* were used as positive controls for RNA quality. Annealing temperatures were optimized for each primer combination. PCR reactions were performed with the primers listed in Table 2. The PCR conditions were as follows: 95°C for 5 min; 35 cycles of 95°C for 15 s, 55–60°C for 30 s and 72°C for 30 s, and 72°C for 10 min. Negative controls containing nuclease free water and total RNA were performed to exclude any DNA contamination. Positive controls containing total genomic DNA from *S. coelicolor* M145 were performed to ensure specific amplification of the PCR product. The PCR products were separated during electrophoresis on 2% agarose gels. All reverse transcription/PCR reactions were carried out in triplicate using RNA isolated from three independent cultivations.

**Table 2:** **Primers used in this study**.

**Oligonucleotide**	**Sequence 5′-3′**	**References**
rt_*hrdB*1 (as control)	GAGTCCGTCTCTGTCATGGCG	Tiffert et al., 2008
rt_*hrdB*2 (as control)	TCGTCCTCGTCGGACAGCACG	
rt_*glnA*1	GGGACAAGACCCTCAACATC	
rt_*glnA*2	CTTGTAGCGGACCTTGTAAC	
rt_*amtB*1	TCCTGGTCTTCCAGCTGATG	
rt_*amtB*2	TTGCCGATGACGAGGATCAC	
rt_*glnII*1	ACCTGGAGAACTGCCTGAAG	
rt_*glnII*2	TGATGATCGCGTCGTAACCC	
rt_*glnR*1	GACGACGTACTGCTCGACAC	
rt_*glnR*2	TCGGCCTTCTCGGACTTATC	
RT_*nirB*fw	GTGCTCGCCCAGCAGTCCGAGC	Amin et al., 2012
RT_*nirB*rev	CCAGCCCCGCCTCCCGCGCCAG	
cobB1fw_NdeI	CATATGGCGCATGCGCCCCACTCTGAG	This work
cobB1rev_BamHI	GGATCCGGCCGTCGCCGCGTCCCCCAC	
cobB2fw_NdeI	CATATGACCGGCAAGCCTCTCGTCGCC	
cobB2rev_BamHI	GGATCCGCCCAGCCCGCGCAGCAGCG	

*Restriction enzymes sites are underlined*.

### Strep-tagged GlnR overexpression in *S. coelicolor* M145 and purification

For Strep-GlnR purification, *S. coelicolor* M145 carrying pGM-Strep-*glnR* overexpression strain (Table 1) was grown for 4 days in complex S-medium at 30°C. Subsequently, cells were harvested and washed twice with nitrogen-free Evans medium. Washed cell biomass was transferred into Evans medium supplemented with 5 or 100 mM NH_4_Cl and 5 or 100 mM NaNO_3_ as a sole nitrogen source, respectively. The expression of Strep-GlnR was induced with 12.5 μg/ml thiostrepton for 36 h. After cultivation cells were harvested and washed with a solution of 100 mM Tris and 150 mM NaCl (pH 8.0). In order to prevent phosphatase and protease activity, 5 mM sodium fluoride and 5 mM orthovanadate and the EDTA-free cOmplete protease inhibitor cocktail (Roche) were added to the buffer. Cell lysis was performed by Emulsifex (Avestin, Ottawa, Canada) with three consecutive passages. Cell debris and insoluble proteins were separated from the soluble fraction by centrifugation (60 min, 14800 g, 4°C). Soluble proteins were loaded onto a pre-equilibrated Gravity flow Strep-Tactin^®;^ Sepharose^®;^ column for one-step purification of recombinant Strep-tag^®;^ proteins (1-ml bed volume; IBA, Germany). Strep-GlnR was competitively eluted using elution buffer supplemented with 2.5 mM desthiobiotin. Concentrated fractions containing the pure Strep-GlnR were stored at 4°C.

### Cloning, overexpression, and purification of the his-tagged CobB1 and CobB2 in *E.coli* BL21

Oligonucleotide primers (Table 2) were designed to incorporate an NdeI and BamHI restriction sites into PCR-fragments containing *cobB1* (*SCO0452*) and *cobB2* (*SCO6464*) genes. The PCR products were cloned into pET15b (Novagen, UK) to generate plasmids pET15b-CobB1 and pET15b-CobB2 used to transform *E. coli* BL21 (DE3). The over-night cultures of *E.coli* BL21 pET15b-CobB1 and *E.coli* BL21 pET15b-CobB2 were used to inoculate 500 ml fresh LB containing 12.5 μg/ml chloramphenicol and 50 μg/ml ampicillin. At an OD_578_ of 0.3, the cells were induced for the expression of His-CobB1 and His-CobB2 with of 0.1 mM IPTG and further incubated at 30°C for 5–10 h. All subsequent procedures were performed at 4°C. Cells were harvested by centrifugation at 5000 rpm for 30 min and resuspended in lysis buffer [50 mM Tris-HCl buffer; pH 7.5; supplemented with the EDTA-free cOmplete protease inhibitor cocktail (Roche)]. Cell lysis was performed by Emulsifex (Avestin, Ottawa, Canada). Cell debris and insoluble proteins were separated from the soluble fraction by centrifugation (30 min, 14,800 g, 4°C). Soluble proteins were loaded onto a preequilibrated Ni^2+^-nitrilotriacetic acid (NTA)-agarose column (1-ml bed volume; IBA, Germany), and His-CobB1 and CobB2 were competitively eluted using elution buffer supplemented with 200 mM imidazole. Fractions containing the pure proteins were pooled and desalted using a HiTrap desalting column (GE Healthcare) with 20 mM Tris-HCl buffer. Concentrated fractions His-CobB1 and His-CobB2 were stored at 4°C.

### Western blot analysis

*S. coelicolor* M145 was grown, harvested and lysed as described above. The protein concentration was determined using a Nanodrop (PEQLAB, Erlangen, Germany). Proteins were separated by SDS-PAGE (Laemmli, 1970) and transferred to a nitrocellulose membrane (Roth, Karlsruhe, Germany) by semidry electroblotting (PEQLAB) using transfer buffer (25 mM Tris, 150 mM glycine, 20% methanol, pH 9.2) for 30 min at 400 mA. The membrane was blocked with TBST buffer with 5% BSA at room temperature for 1 h. Subsequently the membrane was incubated at room temperature for 1 h with rabbit anti-GlnR polyclonal antibodies (SEQLAB, Göttingen, Germany) or overnight at 4°C with rabbit anti-*N*-acetyl lysine polyclonal antibodies (Cell Signaling TECHNOLOGY) in TBST buffer for GlnR detection (10 mM Tris pH 8, 150 mM NaCl, 0.05% Tween 20 supplemented with 2.5% of milk powder) or TBST buffer for *N*-acetyl-lysine detection (25 mM Tris pH 8.0, 125 mM NaCl, 0.1% Tween 20 supplemented with 3% of BSA), respectively. Membranes were washed with TBST (four times) for 5 min and the binding of the primary antibodies against GlnR protein or *N*-acetylated lysine was detected using anti-rabbit IgG horse-radish-peroxidase-conjugated antiserum (BIORAD, München, Germany) solved in TBST buffer. After 2 h of incubation at ambient temperature the binding of secondary antibodies against rabbit antibodies was detected using the ECL Western blotting detection system (GE Healthcare, München, Germany).

### Electrophoretic mobility shift assay (EMSA)

DNA fragments containing promoter regions of GlnR target genes were amplified with Taq polymerase (GENAXXON bioscience, Germany) using genomic DNA from *S. coelicolor* M145. For this, genomic DNA was isolated with the NucleoSpin® Tissue Kit (Macherey-Nagel, Düren, Germany). Primer sequences, PCR and labeling conditions were carried out as reported in Tiffert et al. (2008). All EMSA reactions were carried out in EMSA buffer (100 mM Tris, 150 mM NaCl, 10 mM β-mercaptoethanol, pH 8) containing an excess of unlabeled nonspecific salmon sperm DNA. The Cy5-labeled target DNA (2 ng) and 4 μg of Strep-GlnR protein or 50 μg of the cleared cell lysate from *glnR* mutant were dissolved in EMSA buffer and incubated for 10 min at 24°C. After incubation, loading buffer (0.25 × TBE buffer and 60% glycerol) was added and the fragments were separated using gel electrophoresis on 2% TAE agarose gels. DNA bands were visualized by the fluorescence imaging using a Typhoon Trio+ Variable Mode Imager (GE Healthcare).

### *In vitro* deacetylation of Strep-GlnR

Deacetylation of Strep-GlnR was performed in the presence of NAD^+^ as a substrate for the deacetylase. For this reaction, 100 μg of Strep-GlnR in 50 mM Tris-HCl pH 8.3, were incubated with 1 mM NAD^+^ for 6 h at 30°C in the presence and absence of 20 μg of His-CobB1 or His-CobB2. Acetylated and deacetylated Strep-GlnR samples were analyzed by immunoblotting using rabbit polyclonal anti-*N*-acetyl lysine antibodies (Cell Signaling TECHNOLOGY).

### Nano LC-MS/MS analysis of the purified strep-GlnR

Purified Strep-GlnR was—either in solution or in gel—digested with trypsin (1:100 w/w) as described previously (Borchert et al., 2010). Ten percentage of the peptide mixtures in the resulting in-solution digests were directly analyzed by LC-MS/MS. Additionally, the tryptic peptides were subjected to titanium dioxide chromatography to enrich detection of the phosphorylated peptides. For phosphopeptide enrichment acetonitrile was added to the peptide mixture to a final concentration of 30% and the pH was adjusted to 2–3. Enrichment of phosphopeptides by titanium dioxide chromatography was done as described previously (Olsen and Macek, 2009) with the following modifications: phosphopeptide elution from the beads was performed three times with 100 ml of 40% ammonia hydroxide solution in 60% acetonitrile at a pH > 10.5.

For peptide analysis a Proxeon Easy-LC system (Proxeon Biosystems, Odense, Denmark) coupled to a LTQ-Orbitrap-XL (Thermo Fisher Scientific, Bremen, Germany) equipped with a nanoelectrospray ion source (Proxeon Biosystems) as described previously (Koch et al., 2011) was used. The five most intense precursor ions were fragmented by activation of neutral loss ions at −98, −49, and −32.6 relative to the precursor ion (multistage activation). Mass spectra were analyzed using the software suite MaxQuant, version 1.0.14.3 (Cox et al., 2009). The data were searched against a target-decoy *Streptomyces coelicolor* database containing 8154 forward protein sequences and 262 frequently observed contaminants. Trypsin was set as protease in which two missed cleavage sites were allowed. Beside acetylation at the *N*-terminus of lysine and oxidation of methionine, phosphorylation of serine, threonine, and tyrosine were set as variable modifications. Carbamidomethylation of cysteine was set as fixed modification. Initial precursor mass tolerance was set to 7 parts per million (ppm) at the precursor ion and 0.5 Da at the fragment ion level. Identified peptides were parsed using the identify module of MaxQuant and further processed for statistical validation of identified peptides, modified sites and protein groups. False discovery rates were set to 1% at peptide, modified site, and the protein group level. To assign a phosphorylation and acetylation site, respectively, to a specific residue a minimal reported localization probability of 0.75 was set as a threshold. The fragmentation spectra of potential modified peptides were manually validated for presence of phosphorylation and acetylation sites.

## Results

### Revisiting the transcriptional regulation of *glnR* and GlnR-target genes under defined and complex nitrogen conditions

**Figure 1:**
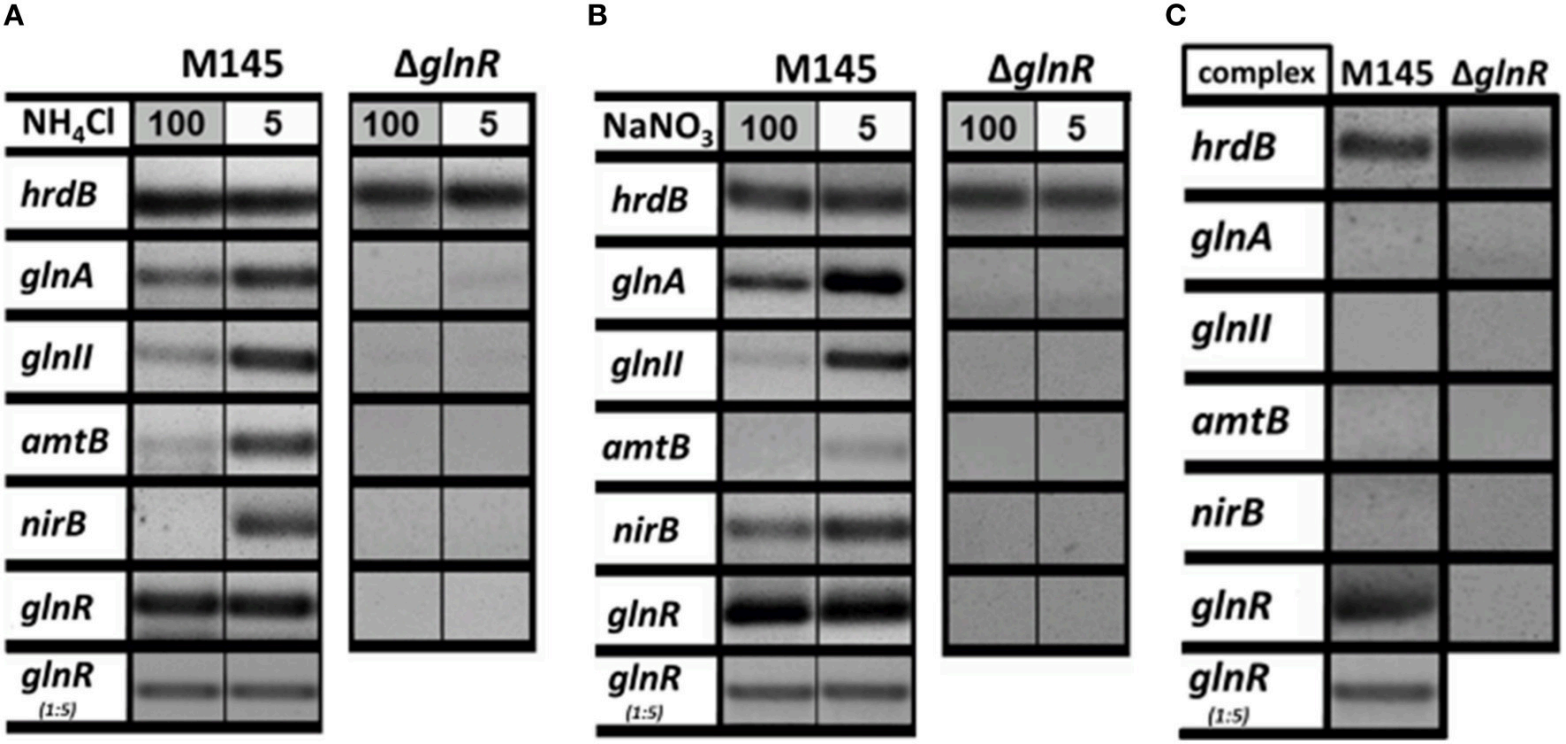
**Transcriptional analysis of the selected GlnR target genes under variable nitrogen and carbon conditions. (A)** RT-PCR of *glnA, glnII, amtB, nirB*, and *glnR* in *S. coelicolor* M145, and *glnR* mutant cultivated in defined Evans medium with low (5 mM) or high (100 mM) concentrations of ammonium chloride as sole nitrogen source. **(B)** RT-PCR of *glnA, glnII, amtB, nirB*, and *glnR* in *S. coelicolor* M145 and *glnR* mutant cultivated in defined Evans medium with low (5 mM) or high (100 mM) sodium nitrate as sole nitrogen source. **(C)** RT-PCR of *glnA, glnII, amtB, nirB*, and *glnR* in *S. coelicolor* M145 and *glnR* mutant cultivated under complex nitrogen rich conditions (S-medium). Total RNA was isolated from mycelium harvested after 24 h of cultivation.

The GlnR regulator controls genes related to *N*-assimilation including genes involved in the ammonium uptake (operon *amtB-glnK-glnD)*, nitrate and nitrite assimilation (*nnaR, nasA, nirB, narK*) and synthesis of the central metabolic nitrogen donors glutamine and glutamate (*glnA, glnII*, and *gdhA*; Tiffert et al., 2008; Amin et al., 2012). Although many genetic studies on the regulation of the *N*-assimilatory genes in *S. coelicolor* and other actinobacteria have been performed, the regulation of the GlnR activity itself is still enigmatic. In a first step, we aimed to show how *glnR* itself and selected GlnR target genes are regulated at the transcriptional level upon growth in four defined *N*-conditions as well as in a complex medium. For this purpose, RT-PCR was performed using total RNA isolated from *S. coelicolor* M145 and the *glnR* mutant. The strains were grown for 4 days in the complex S-medium to obtain high biomass. Cells were harvested by centrifugation and washed with Evans medium to remove traces of S-medium. Subsequently the biomass was transferred into defined Evans medium containing different *N*-sources (ammonium chloride or sodium nitrate) at 100 mM (*N*-excess) or 5 mM (*N*-limitation) or into complex S-medium. All cultures were further cultivated for 24 h, at 30°C. Total RNA isolated from *S. coelicolor* M145 and the *glnR* mutant was used to generate cDNA. Subsequently, RT-PCR analysis using internal primers for *glnR* and selected GlnR target genes was performed. The *hrdB* (encoding the essential principal sigma factor of RNA polymerase) was used as an internal standard due to its relatively constant levels of expression throughout the growth (Buttner et al., 1990). All reverse transcription/PCR reactions were carried out in triplicate using RNA isolated from three independent cultures (for details see Section RT-PCR). For the transcriptional analysis the following GlnR target genes encoding proteins involved in ammonium and nitrate assimilation were selected: *glnA, glnII* (encoding glutamine synthetase GSI and GSII, respectively), *amtB* (encoding an ammonium transporter), and *nirB* (encoding a nitrate reductase). The transcriptional analysis confirmed that GlnR enhanced the expression of *glnA, glnII, amtB*, and *nirB* under low concentration of ammonium chloride, whereas high ammonium chloride concentration inhibited expression of these genes (Figure 1A). To verify whether this regulatory effect was ammonium chloride dependent, transcript levels for the selected GlnR target genes were also analyzed in the presence of sodium nitrate. Our results showed that expression of all tested genes *glnA, glnII, amtB*, and *nirB* was strongly induced in condition of low nitrate concentration in *S. coelicolor* M145 (Figure 1B). At last, transcriptional analysis revealed that the *glnA, glnII, amtB*, and *nirB* were not expressed in *S. coelicolor* M145 grown in S-medium (Figure 1C). Expression of *glnA, glnII, amtB*, and *nirB* was totally abolished in the *glnR* mutant under all tested conditions, indicating that GlnR was necessary for their expression. These results indicate that expression of the selected GlnR target genes was strictly regulated by GlnR whose activity seemed to be modulated according to the *N*-status of the cell. Transcriptional analysis showed that the *glnR* transcript was present under all tested conditions, demonstrating that *glnR* is not regulated at the transcriptional level in the response to changing *N*-concentrations. These analyses led us to assume that GlnR regulatory activity might be modulated by post-translational modifications.

### The GlnR protein is present in the cell under both *N*-limited and *N*-proficient conditions

**Figure 2:**
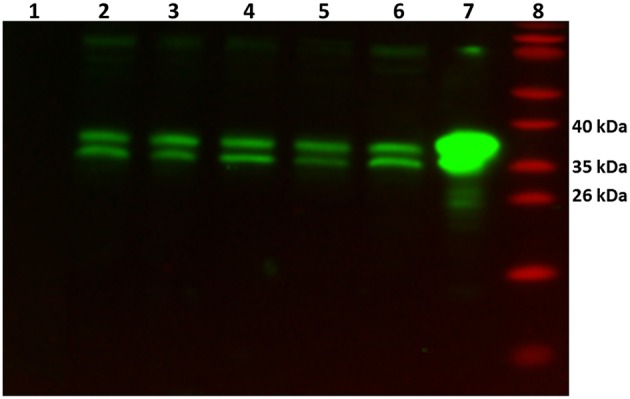
**Detection of the native GlnR protein in the cell lysate from *S. coelicolor* M145 cultivated under different defined nitrogen conditions**. Western blot analysis of GlnR probed with anti-GlnR antibodies generated in rabbit (1:5000) and the secondary goat anti-rabbit antibodies conjugated with HRP (1:3000). Lane 1: cell lysate from *glnR* mutant grown in S-medium (200 μg of total protein)—negative control, Lane 2–6: *S. coelicolor* M145 cell lysates (200 μg of total protein, each). Cell lysates generated from cells grown in complex S-medium (Lane 2), in defined Evans medium with 5 mM NH_4_Cl (Lane 3), 100 mM NH_4_Cl (Lane 4), 5 mM NaNO_3_ (Lane 5), and 100 mM NaNO_3_ (Lane 6). Lane 7: cell lysate from the Strep-GlnR overexpression strain grown in S-medium—positive control. Lane 8: PageRuler Prestained Protein Ladder (Fermentas). Location of the GlnR with the estimated molecular masses of ~35 and 38 kDa.

In order to verify whether the GlnR protein was present (as its transcript) in all tested conditions, Western blot analysis was performed. As a control cell lysates from the *glnR* mutant as well as purified Strep-GlnR protein were used. For this, *S. coelicolor* M145 was grown in S-medium for 4 days at 30°C; cells were washed twice with Evans medium to remove traces of the complex S-medium. The biomass was transferred into defined Evans medium and further cultivated for 36 h at 30°C*. S. coelicolor* M145 cells from S-medium and Evans medium were harvested and disrupted. As a negative control cell lysate generated from the *glnR* mutant grown in S-medium was used. As a positive control Strep-GlnR overexpressed and purified from the *S. coelicolor* M145 grown in S-medium was used. Clarified cell lysates (200 μg of total protein) as well as isolated Strep-GlnR protein (20 μg) were run on a 12.5% SDS polyacrylamide gel and subsequently transferred onto a nitrocellulose membrane. Signals for the native GlnR and Strep-GlnR were detected by using anti-GlnR antibodies. Western blot analysis revealed the presence of native GlnR in *S. coelicolor* M145 under all tested conditions, whereas no signal was detected in the cell lysate from the *glnR* mutant. No sign of GlnR proteolysis could be detected under *N*-limitation or proficiency. The predicted size of the GlnR protein calculated from the amino acid sequence is 29.8 kDa. Interestingly, GlnR appeared as a double band with the estimated size of ~35 and 38 kDa (Figure 2). This indicated that this regulator may undergo post-translational modification.

### Only GlnR interacts with GlnR-target genes

In order to determine whether other regulators besides GlnR other regulators are able to recognize and interact with selected promoters of the GlnR-target genes, comparative EMSAs were performed using cell lysates from the *glnR* mutant and Cy5-labeled promoter regions of P_*glnA*_, P_*amtB*_, and P_*glnII*_. To do so, the *glnR* mutant was grown in S-medium for 4 days at 30°C; cells were washed twice with Evans medium, transferred into Evans medium or directly transferred in S-medium and further cultivated for 36 h at 30°C. Cells were harvested, disrupted and the cell lysates (50 μg of total protein) were used for EMSAs. No shifts were observed with cell lysates generated from the *glnR* mutant (Figure S1), indicating that solely GlnR was able to recognize tested promoters under studied conditions.

### Different GlnR post-translational modification patterns were detected under complex *N*-Rich conditions and *N*-Defined conditions

Post-translational modifications of GlnR were identified by LC-MS/MS using Strep-GlnR overexpressed and isolated from *S. coelicolor* M145 grown in the complex and *N*-rich S-medium. The purified Strep-GlnR samples were run on a 12.5% SDS polyacrylamide gel and subsequently stained with Coomassie blue. Finally, the band corresponding to Strep-GlnR was cut out from the gel and subjected to proteolytic digestion with trypsin. In addition to a direct measurement of tryptic Strep-GlnR peptides, phosphopeptides were selectively enriched using titanium dioxide affinity chromatography prior to LC-MS/MS analysis. To specifically detect peptides carrying phosphorylated and acetylated residues, the LC-MS/MS spectra of the modified peptides were compared to the intensities of spectra of the corresponding non-modified peptides. Phosphorylation and acetylation sites were considered as resulting from high confidence phosphorylation and acetylation events only if the LP was higher to 0.75 (or equal), the PEP score was lower than 0.01 (or equal) and the Mascot score higher than 39 (or equal; for details of this evaluation see Materials and Methods, in the Section Nano LC-MS/MS Analysis of the Purified Strep-GlnR). Mapping of the post-translational modifications on the Strep-GlnR purified from *S. coelicolor* M145 grown in this medium revealed peptides carrying phosphorylated serine and threonine residues: Ser 133, Thr 138, Ser 207, Thr 211, Thr 256, Ser 264/265, and only one acetylated residue, Lys 142 (Table 3 and Supplementary Material Data Sheets 1–4).

**Table 3:** **Phosphorylated and acetylated GlnR peptides detected by LC-MS/MS under complex and defined *N*-conditions**.

**Modified GlnR peptides (nitrogen rich, complex S-medium)**	**Phosphosite (ph) and acetylation site (ac)**	**LP**	**PEP**	**MASCOT**
NGDL**S**VDEATYSAK	(ph) Ser 133	0.999	0.000	49.52
NGDLSVDEA**T**YSAK	(ph) Thr 138	0.999	0.000	49.52
LGPEHE**S**LIGTVR	(ph) Ser 207	0.970	0.000	44.29
LGPEHESLIG**T**VR	(ph) Thr 211	0.999	0.000	44.29
AAAE**T**NEAAGAR	(ph) Thr 256	1.000	0.000	37.80
AAAETNEAAGAR**SS**KV	(ph) Ser 264/265	0.979/0.999	0.000	37.80
NGDLSVDEATYSA**K**	(ac) Lys 142	1.000	0.000	60.27
**MODIFIED GlnR PEPTIDES (DEFINED EVANS MEDIUM WITH 100 mM NaNO_3_)**				
NGDLSVDEA**T**Y**S**AK	(ph) Ser 140	0.999	0.000	54.70
AAAE**T**NEAAGAR	(ph) Thr 256	1.000	0.001	42.16
NGDLSVDEATYSA**K**	(ac) Lys 142	0.999	0.000	66.18
VLDLTF**K**EFELLK	(ac) Lys 153	1.000	0.000	86.48
VLDLTFKEFELL**K**YLAQHPGR	(ac) Lys 159	1.000	0.000	68.96
A**K**LGPEHESLIGTVR	(ac) Lys 200	1.000	0.000	86.58
**MODIFIED GlnR PEPTIDES (DEFINED EVANS MEDIUM WITH 5 mM NaNO_3_)**				
NGDLSVDEATYSA**K**	(ac) Lys 142	0.999	0.000	103.18
VLDLTF**K**EFELLK	(ac) Lys 153	1.000	0.000	76.10
VLDLTFKEFELL**K**YLAQHPGR	(ac) Lys 159	1.000	0.000	76.10
A**K**LGPEHESLIGTVR	(ac) Lys 200	1.000	0.000	86.56

*Acetylated or phosphorylated residues are in bold*.

Post-translational modifications of GlnR under defined *N*-conditions were identified by LC-MS/MS using Strep-GlnR isolated from *S. coelicolor* M145 grown in Evans media supplemented with either 100 mM NaNO_3_ (nitrate proficiency) or 5 mM NaNO_3_ (nitrate limitation) as a sole *N*-source. Preparation of the Strep-GlnR samples, LC-MS/MS analysis and data processing were performed as stated in the Section Different GlnR Post-translational Modification Patterns Were Detected under Complex *N*-Rich Conditions and *N*-Defined Conditions. Strep-GlnR isolated from cells grown under nitrate proficient conditions revealed only two phosphorylated residues: Ser 140 and Thr 256, whereas no serine/threonine phosphorylation was detected in Strep-GlnR isolated from cells grown under nitrate limitation. Interestingly, Strep-GlnR isolated from *S. coelicolor* M145 cultivated in condition of nitrate proficiency or limitation exhibited identical acetylation pattern (Table 3 and Supplementary Material Data Sheets 1–4). The following lysine residues were acetylated in Strep-GlnR independent of the nitrate concentration in the Evans medium: Lys 142, Lys 153, Lys 159, and Lys 200, whereas only Lys142 was acetylated when Strep-GlnR originated from culture in S-medium. The Strep-GlnR isolated from cells grown in defined Evans medium supplemented with nitrate as a sole *N*-source revealed higher acetylation level than the Strep-GlnR from complex S-medium. Most of the acetylated and phosphorylated GlnR residues were localized within the helix turn helix motif involved in the DNA recognition and binding. These modifications are thus likely to have an impact on the GlnR DNA-binding activity.

### Serine/threonine phosphorylation influenced the DNA-binding activity of GlnR

**Figure 3:**
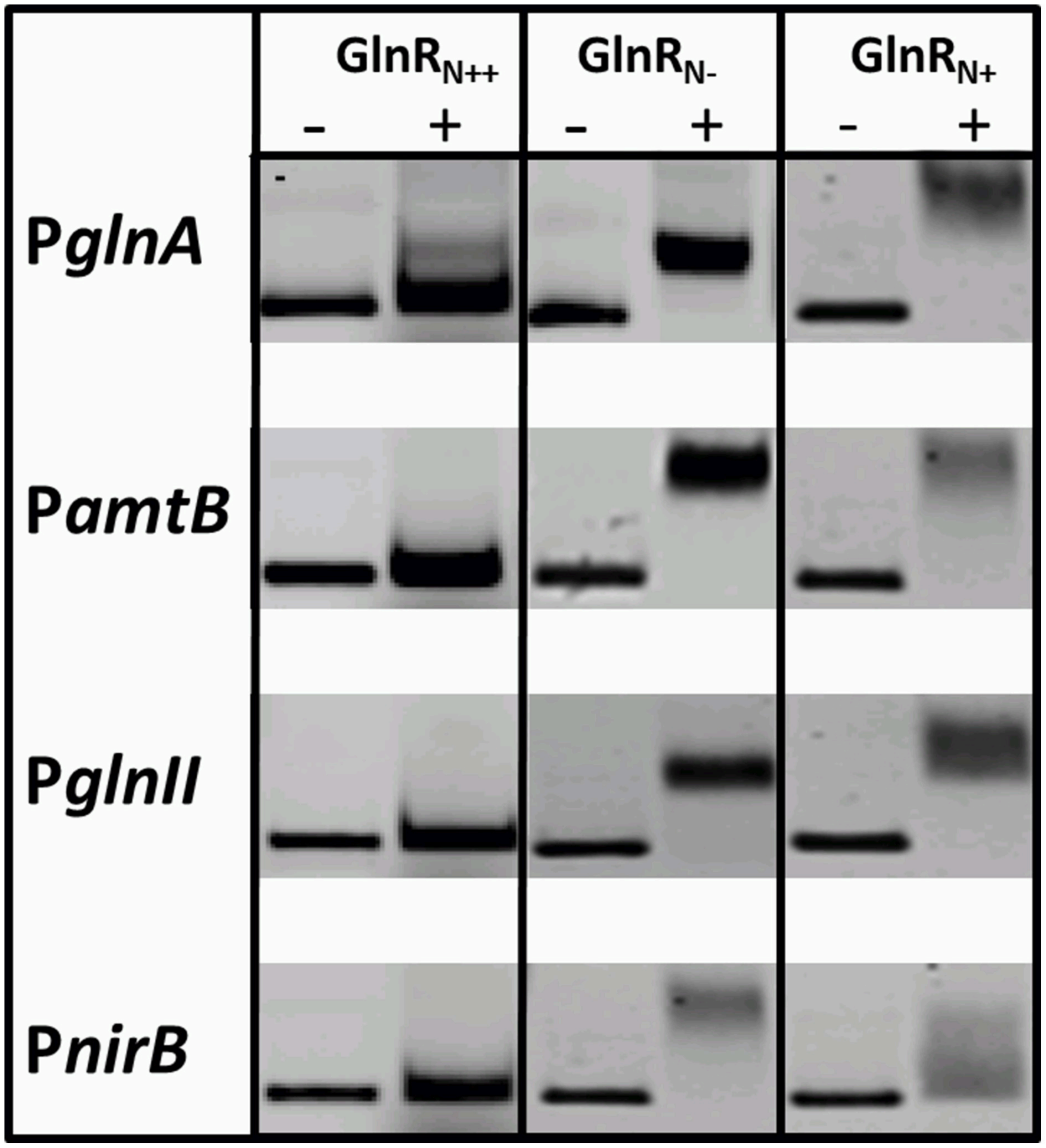
**EMSAs demonstrating different binding behavior of Strep-GlnR depending on differential modification patterns**. Lane (−), control, promoter regions of the known GlnR target genes (Cy5—labeled probe without Strep-GlnR protein). Lane (+), Cy5—labeled probe with 4 μg of the Strep-GlnR_N+_ from complex, rich condition (phosphorylated on the six Ser/Thr residues and acetylated on the one Lys residue), Strep-GlnR_N−_from nitrate limited conditions (acetylated on four Lys residues), and Strep-GlnR_N+_ from the nitrate excess conditions (acetylated on four Lys residues and additionally phosphorylated on two Ser/Thr residues). The differential modification pattern of Strep-GlnR was confirmed by LC-MS/MS prior EMSAs. EMSAs were performed in the presence of the 300 × excess of the unlabeled, unspecific salmon sperm DNA.

GlnR was previously shown to specifically bind numerous promoters of genes that encode proteins involved in *N*-assimilation in *S. coelicolor* M145. GlnR binding boxes were defined and localized in the promoter regions of *glnA, glnII, amtB, nirB*, and other genes encoding proteins involved in *N*-metabolism (Tiffert et al., 2008; Amin et al., 2012). In order to determine whether the differently modified GlnR protein isolated from *S. coelicolor* M145 could recognize and interact with the selected target promoters comparative electrophoretic mobility shift assays (EMSAs) were performed using the Cy5-labeled promoter regions P_*glnA*_, P_*amtB*_, P_*glnII*_, P_*nirB*_. Three different modified forms of Strep-GlnR isolated from *S. coelicolor* M145 grown either under complex nitrogen rich conditions (Strep-GlnR_N++_) or nitrogen defined conditions (nitrate limited—Strep-GlnR_N−_; nitrate excess—Strep-GlnR_N+_) were used. Post-translational modifications of Strep-GlnR_N++_, Strep-GlnR_N−_, and Strep-GlnR_N+_ were confirmed by LC-MS/MS prior to EMSAs analysis. Multiply phosphorylated Strep-GlnR_N++_ was not able to interact with the promoter regions P_*glnA*_, P_*amtB*_, P_*glnII*_, and P_*nirB*_ indicating that phosphorylation inhibits the binding of Strep-GlnR_N++_ to the target DNA. Interestingly, multiply acetylated GlnR (Strep-GlnR_N−_) was able to interact with and to shift all tested promoter regions. Finally, the multiply acetylated Strep-GlnR_N+_ also phosphorylated on the Ser 140 and Thr 256 residues, generated diffuse shifts with P_*glnA*_, P_*amtB*_, P_*glnII*_, and P_*nirB*_, suggesting different binding of the GlnR or instability of the GlnR-DNA complex (Figure 3). The Ser/Thr phosphorylations altered the *in vitro* DNA-binding activity of GlnR purified from cells grown under *N*-excess conditions while the multiple acetylations did not inhibit the formation of GlnR-DNA complexes under *N*-limiting conditions. These results are consistent with the transcriptional analysis of the GlnR-target genes under *N*-limited and excess conditions reported in *Results Revisiting the Transcriptional Regulation of glnR and GlnR-Target Genes under Defined and Complex Nitrogen Conditions*. High induction of the expression of GlnR target genes under nitrogen limitation is thus achieved by the unphosphorylated GlnR as shown by the LC-MS/MS analysis.

### Impact of the acetylation of GlnR on its DNA-binding activity

**Figure 4:**
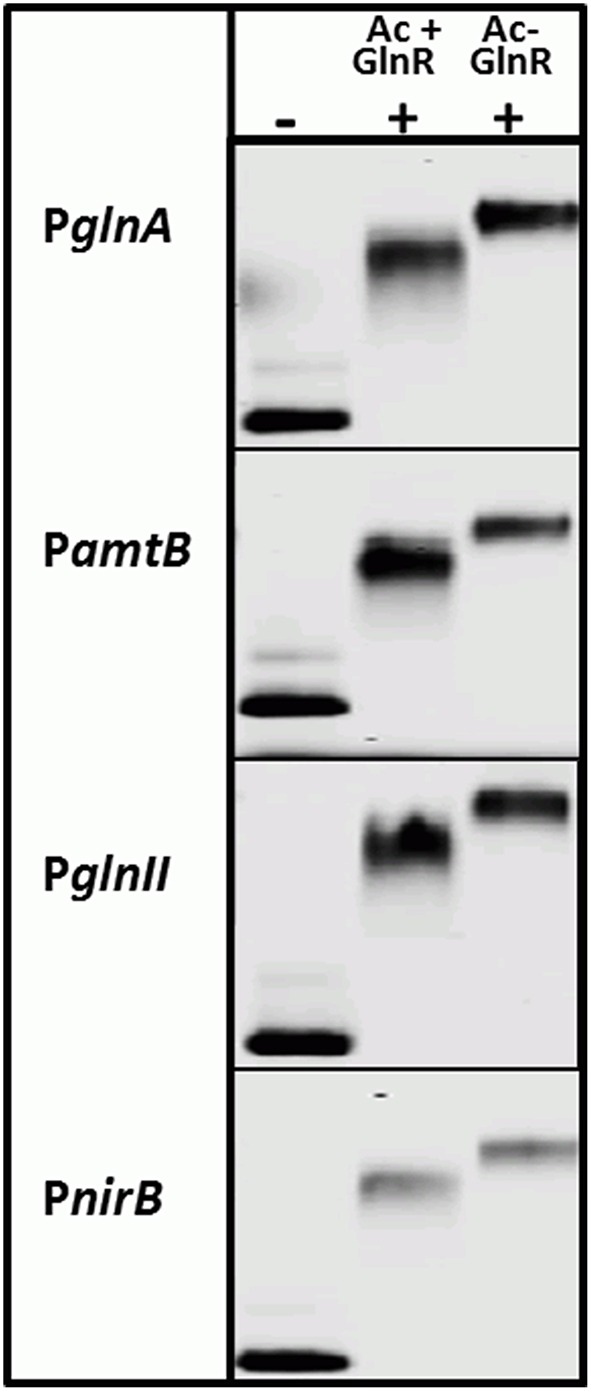
**EMSAs demonstrating different binding behavior of the acetylated Strep-GlnR and *in vitro* deacetylated Strep-GlnR**. Lane (−), control, promoter regions of the known GlnR target genes (Cy5—labeled probe without Strep-GlnR protein). Lane (+), Cy5—labeled probe with 4 μg of the acetylated Strep-GlnR (Ac+) or deacetylated Strep-GlnR (Ac−). The loss of the acetylation on Strep-GlnR was confirmed by Western blot analysis prior EMSAs. EMSAs were performed in the presence of the 300 × excess of the unlabeled, unspecific salmon sperm DNA.

Interestingly, the Ser/Thr phosphorylation of GlnR occurs in condition of *N*-proficiency whereas acetylation is independent of the *N*-status of the cell. To study the impact of the acetylation on the GlnR DNA-binding activity, we attempted to remove the acetyl groups from GlnR by an enzymatic deacetylation. *S. coelicolor* M145 possess two genes (*SCO0452* and *SCO6464*) encoding enzymes annotated as a sirtuin-like (deacetylase-like). SCO0452 named CobB1 is most similar to human sirtuin SIRT4 and was functionally characterized as NAD^+^-dependent deacetylase from *S. coelicolor* (Mikulik et al., 2012). The SCO6464 designated as CobB2, shares significant homology with human SIRT5 (Moore et al., 2012). The CobB2 homolog in *S. erythraea* (with 68% similarity on the protein level) was shown to catalyze deacetylation of acetyl-CoA synthetase AcsA *in vitro* (You et al., 2014). Bacterial sirtuins are able to deacetylate a large number of target proteins (Castaño-Cerezo et al., 2014) and shows no preference for enzymatic and nonenzymatic lysine acetylation substrate sites (AbouElfetouh et al., 2014). We therefore assumed that the CobB1 and CobB2 deacetylases from *S. coelicolor* might be potentially able to deacetylate GlnR. Overexpression of *N*-terminally His_6_-tagged CobB1 and CobB2 (His-CobB1 and His-CobB2) was achieved with the IPTG-inducible system in *E.coli* BL21. Acetylated Strep-GlnR, isolated from *S. coelicolor* M145 grown under *N*-limiting conditions was used as a substrate for the *in vitro* deacetylation assays. A successful GlnR deacetylation was proved by Western blot analysis. Signals for acetylated Strep-GlnR (Ac+) protein and deacetylated Strep-GlnR (Ac−) were detected by anti-GlnR antibodies and anti-*N*-acetyl lysine antibodies. Western blot analysis revealed that the deacetylase CobB2 was able to remove the acetyl groups from Strep-GlnR in the *in vitro* assay (Figure S2). EMSAs performed with double-stranded Cy5-labeled selected promoter regions of P_*glnA*_, P_*amtB*_, P_*glnII*_, and P_*nirB*_ showed that both the Strep-GlnR (Ac−) as well as the Strep-GlnR (Ac+) were able to interact with the target DNA (Figure 4). However, the Strep-GlnR (Ac+) generated more retarded shift than did the deacetylated Strep-GlnR (Ac−), suggesting that acetylation changes the DNA-binding affinity of GlnR.

## Discussion

Members of the family of *Streptomycetaceae* are well-known antibiotic producers. The production of antimicrobial compounds ensured the adaptation of these bacteria to a wide variety of ecological niches and successful competition with other microorganisms for space and resources in their soil habitat. Depending on the soil type and seasonal changes, soil may exhibit a high diversity in nutrients availability ranging from nutrient-poor to nutrient-rich conditions. Streptomycetes are able to adjust rapidly to these changing conditions. To do so, sensing, and responding to changes in a nutrient availability and subsequently coordination of the metabolic switch is necessary. The global nitrogen response regulator GlnR in *S. coelicolor* controls genes related to *N*-metabolism and ensures a dynamic and fast response under fluctuating *N*-conditions (Tiffert et al., 2008; Amin et al., 2012). Our transcriptional analysis revealed strong expression of *glnR* under all tested conditions. This indicates that *glnR* expression is not regulated at the transcriptional level in the response to the *N*-availability in *S. coelicolor* M145 as in other members of *Actinomycetales* such as: *M. smegmatis* (Amon et al., 2008; Petridis et al., 2015), *A. mediterranei* U32 (Wang et al., 2014) and *Microbispora* ATCC-PTA-5024 (to be published). The transcriptional regulation of the *glnR* in *S. coelicolor* and *M. smegmatis* is probably not achieved by GlnR itself, since GlnR binding boxes were not detected in the *glnR* promoter region (Tiffert et al., 2008; Jenkins et al., 2013). In contrast, GlnR-self-regulation at the transcriptional level was reported in *S. erythraea* (Yao et al., 2014). Despite its unchanged expression, GlnR controls the expression of its target genes in response to *N*-availability in *S. coelicolor* as well as in *M. smegmatis, A. mediterranei*, and *S. erythraea* (Tiffert et al., 2008; Jenkins et al., 2013; Yao et al., 2014). Indeed, in *S. coelicolor* expression of *glnA, glnII, amtB*, and *nirB* was totally abolished in the *glnR* mutant but it was enhanced in condition of nitrate and ammonium limitation and reduced or completely abolished in condition of *N*-proficiency. The *nirB*, that is expressed at the similar level in the presence of high and low *N*, escapes this general rule likely because GlnR cooperate with NnaR (transcriptional regulator for nitrate/nitrite assimilatory genes, GlnR-target) to regulate *nirB* expression in the presence of nitrate (Amin et al., 2012). These findings led us to assume that GlnR undergoes post-translational modifications that might alter its DNA-binding ability in a response to *N*-availability. Our LC-MS/MS analysis revealed that GlnR was post-translational modified by Ser/Thr phosphorylation and acetylation on Lys residues. Such modifications have not been reported for GlnR in *Actinomycetales* so far. Phosphorylated GlnR was detected in cells grown in the *N*-rich Evans medium and complex S-medium, demonstrating that the phosphorylation of the GlnR regulator is associated with *N*-excess. In contrast, the unphosphorylated form of GlnR was detected by the LC-MS/MS analysis only under *N*-limited conditions. Lack of the phosphorylation on the Asp 50 and lack of any Ser/Thr phosphorylation in GlnR isolated from *S. coelicolor* grown under *N*-limiting conditions was also demonstrated by Lin et al. (2014), in agreement with our results.

It has long been thought that signal transduction systems in bacteria relied solely on histidine/aspartate phosphorylation, while signal transduction systems based on serine/threonine phosphorylation and *N*-lysine acetylation were restricted to eukaryotes. However, with the increasing availability of phosphoproteomic and acetylproteomic data, a number of proteins modified by acetylation and phosphorylation have been detected in bacteria (Soufi et al., 2012; Cain et al., 2014). These post-translational modifications (PTMs) are known to influence changes in the profile of the bacterial transcriptome and proteome. PTMs have significant influence on the protein charge, size, hydrophobicity, and conformation. Therefore, PTMs can alter the activity, stability or cellular location and/or affinity of the modified protein for its binding partners (Hu et al., 2010; Jones and O'Connor, 2011). Large number of proteins phosphorylated on serine and/or threonine residues was identified in *S. coelicolor* (Parker et al., 2010; Manteca et al., 2011), *M. tuberculosis* (Prisic et al., 2010), and other bacteria (see Cain et al., 2014 for review). Some bacterial transcription regulators were reported to be phosphorylated on serine and threonine residues (see Kalantari, 2015 for review). For instance, the post-translational serine/threonine phosphorylation was reported for EmbR, the transcriptional activator of arabinan biosynthesis genes in *Mycobacterium tuberculosis* (Molle et al., 2003; Sharma et al., 2006) and AfsR, the transcriptional activator of *afsS* involved in the regulation of secondary metabolism in *Streptomyces coelicolor* (Sawai et al., 2004).

Alignment of the amino acid sequences of GlnR from *S. coelicolor, A. mediterranei*, and *M. smegmatis* showed that residues corresponding to the phosphorylated Thr 138, Ser 140, and Thr 211 were conserved (Figure S3). Therefore, one can predict that these GlnR residues could be also phosphorylated under *N*-excess conditions in *M. smegmatis* and *A. mediterranei*. Since the GlnR structure was only partially elucidated (only *N* -terminal receiver domain), superimposition of the C-terminal GlnR response domain model on the crystal structure of the DNA-bound response regulator PhoP from *M. tuberculosis* (PDB: 5ed4; He et al., 2016) was performed. This comparative analysis revealed that Thr 138 and Ser 140 correspond to the Thr162 and Glu164 residues in the PhoP crystal structure (He et al., 2016), respectively.

These residues are involved in the hydrophobic interactions stabilizing the PhoP dimer (He et al., 2016). Thus, one can assume that phosphorylation of the corresponding residues in GlnR could influence GlnR dimer formation. The Thr 211 residue from GlnR corresponds to the Thr235 residue in the PhoP structure. The side chain of the Thr 235 residue forms hydrogen bonds with the phosphate in the DNA thereby supporting the binding of the PhoP to the minor groove of the DNA (He et al., 2016). Again one can assume that phosphorylation of the corresponding Thr 211 residue in GlnR could influence its DNA binding affinity. However, solving the full GlnR crystal structure is necessary to achieve a detailed analysis of the influence of the modified residues on GlnR conformation DNA binding ability. Even though, the Ser/Thr phosphorylation has never been reported for GlnR, the post-translational serine/threonine phosphorylation is not uncommon in *S. coelicolor*. Forty proteins involved in gene regulation, central metabolism, protein biosynthesis, membrane transport, cell division, sporulation, and morphological differentiation were reported to be phosphorylated on serine and threonine residues in *S. coelicolor* (Parker et al., 2010; Manteca et al., 2011; Ladwig et al., 2015). The phosphoproteomic studies did not report phosphorylation of GlnR, presumably due to different cultivation conditions used (R5 medium and solid GYM medium Parker et al., 2010; Manteca et al., 2011, respectively).

Lysine acetylation of proteins was first discovered in eukaryotes and best characterized for histones (Waterborg, 2001) and eukaryotic transcription factors (Boyes et al., 1998; Marzio et al., 2000; Furia et al., 2002). Detailed analysis of acetylomes of *E. coli, S. enterica*, and *M. pneumoniae* gave a first wide view on this post-translational modification in bacteria (Yu and Auwerx, 2009; Zhang et al., 2009; Wang et al., 2010; Noort et al., 2012). Recently, the lysine acetylation was also reported for actinobacteria such as: *S. roseosporus* (Liao et al., 2015), *M. tuberculosis* (Liu et al., 2014), and *S. erythraea* (Huang et al., 2015). However, the physiological meaning of lysine acetylation was described only for a few prokaryotic proteins. For example, acetylation blocks the enzymatic activity of the acetyl-coenzyme A synthetase in *Salmonella enterica* (Starai et al., 2002), *Bacillus subtilis* (Gardner et al., 2006), *Rhodopseudomonas palustris* (Crosby et al., 2010), *Mycobacterium smegmatis* (Xu et al., 2011), and *S. coelicolor* (Mikulik et al., 2012), demonstrating conserved regulatory mechanism. The acetylation dependent modulation of the DNA-binding activity of the transcriptional regulator was reported for the capsule and flagellum biosynthesis regulator RcsA in *E. coli* (Thao et al., 2010). Multiply acetylation was also shown to inhibit the protein–protein interactions between the CheY (chemotaxis response regulator) and its target proteins in *E. coli* (Liarzi et al., 2010).

Both serine/threonine phosphorylation and lysine acetylation are conserved throughout evolution in all three life kingdoms: prokaryotes, archea, and eukaryotes (Kennelly, 2002; Choudhary et al., 2009; Thao and Escalante-Semerena, 2011). Many proteins involved in central metabolic processes (synthesis of acetyl-CoA, glycolysis, gluconeogenesis, the TCA cycle and the glyoxylate bypass, glycogen biosynthesis, amino acid biosynthesis, fatty acid metabolism, and urea detoxification) were reported to be *N*-acetylated on lysine residues (Kim et al., 2006; Yu and Auwerx, 2009; Zhang et al., 2009; Wang et al., 2010). Recent reports on acetylation of proteins in bacteria demonstrated its role in the physiological adaptations to changes in carbon nutrient availability as reported for *B. subtilis* (Kosono et al., 2015), *S. enterica* (Wang et al., 2010), and *E. coli* (Weinert et al., 2013). The similar acetylation pattern of GlnR under defined conditions detected by LC-MS/MS was independent of the nitrogen source concentration, demonstrating that acetylation apparently does not reflect the nitrogen status of the cell but might signal carbon source availability as in other microorganisms. Comparative analysis of the amino acid sequences of 37 GlnR homologs from *Streptomyces* sp., revealed overall highly conserved sequence except last 30 residues from the *C*-terminus. Four acetylatable lysine residues Lys 142, Lys 153, Lys 159, and Lys 200 residues were conserved in all GlnR homologs (Figure S4). Superimposing of the GlnR response domain on the DNA-bound PhoP revealed that residues corresponding to acetylated Lys 153 and Lys200 residues are located within the conserved α8 helix involved in the recognition and binding of the target DNA. For instance, the Lys 153 residue in GlnR corresponds to the Thr 177 residue in PhoP (He et al., 2016). The Thr 177 forms a hydrogen bond with the phosphate from the major groove of the PhoP target DNA (He et al., 2016). This interaction (including other interactions resulting from α8 helix) contributes to the binding affinity and influence sequence-specific interactions by changing the conformation of the PhoP, target-DNA, or both (He et al., 2016). Therefore, one could imagine that the acetylation of the corresponding residue Lys 153 in GlnR changes the positive charge of Lys 153 into neutral charge and thus may influence the GlnR DNA-binding affinity. The Lys 200 residue in GlnR corresponds to the Lys 224 residue in PhoP that is located in the C-terminus of the α8 helix close to the Arg 222 and Arg 223 both involved in the interactions with phosphates in the major groove of the DNA. However, these structural comparisons could be seen as speculative and solving the DNA-bound GlnR structure is necessary to determine how modified/unmodified residues participate in the GlnR dimer formation and its DNA-binding ability.

**Figure 5:**
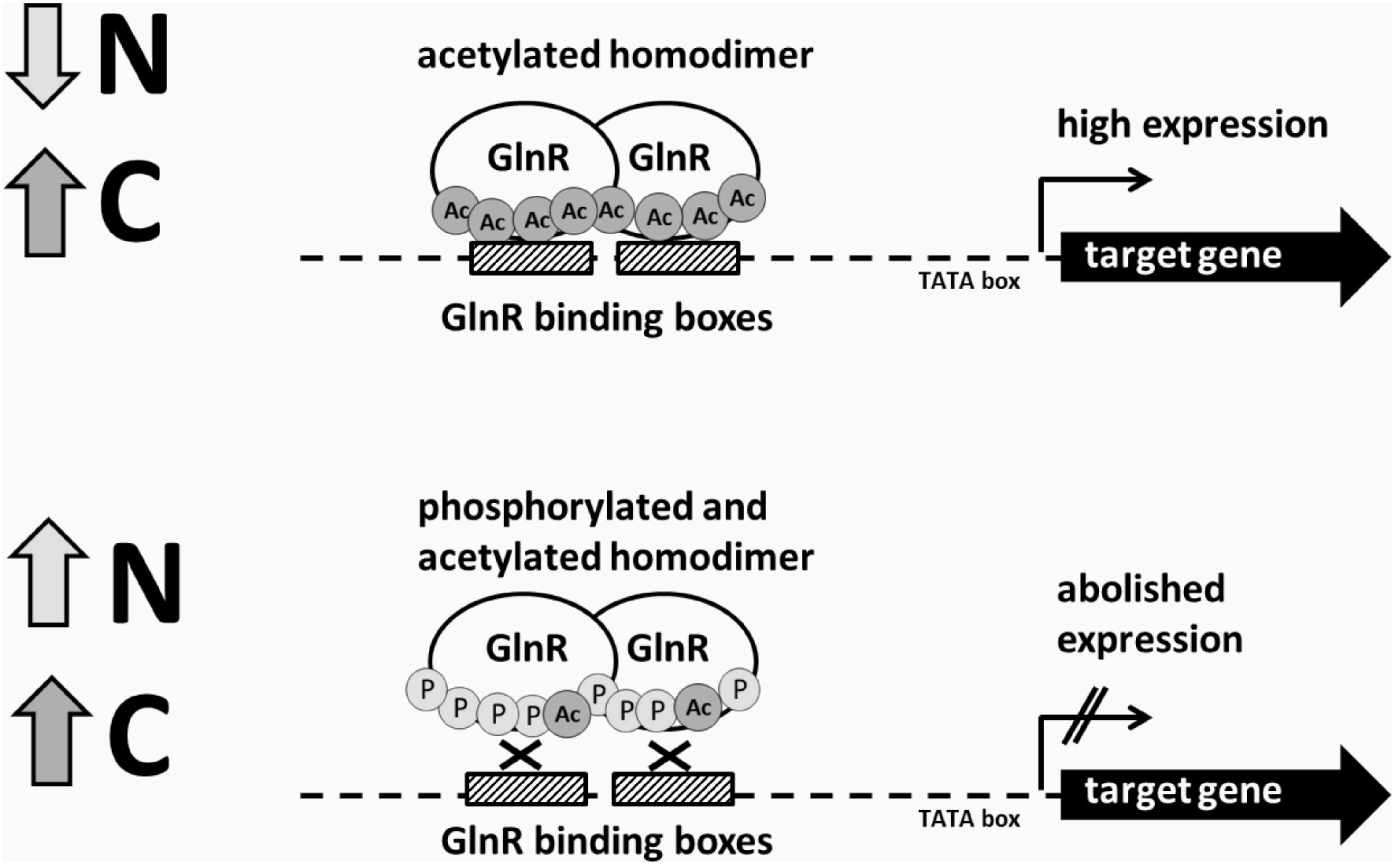
**Schematic model of the regulation of the GlnR-target genes under *N*-rich and *N*-limited conditions by the differentially modified GlnR regulator**.

So far, EMSAs studies were carried out with GlnR from *A. mediterranei* (Wang Y. et al., 2013), *S. erythrea* (Yao et al., 2014), *M. smegmatis* (Jenkins et al., 2013), and *M. tuberculosis* (Malm et al., 2009) overexpressed and purified from *E. coli* and not from the original host. Our study is the first report on changes in the GlnR post-translational modifications and DNA-binding ability in its original host grown under biologically relevant conditions. We could demonstrate that both Ser/Thr phosphorylation and Lys acetylation influenced the DNA-binding activity of GlnR *in vitro*. The influence of the post-translational modifications on the GlnR regulatory function is summarized in the GlnR regulatory model (Figure 5). Bacterial protein modification by acetylation appears to be as common as phosphorylation and often both modifications can be detected on the same protein, suggesting possible cross-talk between these post-translational modifications that might signal *C* and *N* availability (Soufi et al., 2012). Different combinations in the acetylation and phosphorylation events open multitude of possibilities for the regulation of the GlnR regulon depending on the concentration of *N*- but also likely *C*-source in the environment. Our work provides a basis for further studies and detailed analysis of the mechanisms by which *S. coelicolor* senses and responds to changes in nutrient availability and how does GlnR coordinate the regulation of the *N*-related genes to govern the metabolic switch thereby guaranteeing cell homeostasis. In this respect, future work will focus on the identification of the Ser/Thr kinase(s) and acetyltransferase(s) involved in the post-translational modifications of GlnR.

## Author contributions

MHE and MM performed the RT-PCR analysis. RA, YT and MHE performed the EMSAs. YA and SK performed Western blot. RA and MJ overexpressed and purified Strep-GlnR for the LC-MS/MS analysis. MF performed the LC-MS/MS analysis, collected and processed the LC-MS/MS data. JM constructed the His-CobB1 and His-CobB2 overexpression strains and established protein purification method. AM overexpressed and purified His-CobB1 and His-CobB2 necessary for the deacetylation assay and MHE and MM performed deacetylation assay. MHI performed the bioinformatic analysis. NO was involved in the technical assistance. AB formulated the original problem and provided direction and guidance as well as designed the study and developed the methodology. WW and BM provided helpful feedback on an early draft of the paper and assisted with data analysis. AB contributed to the writing of the manuscript and resolved final approval of the version to be published.

## Funding

RA was funded by a scholarship provided by the Higher Education Commission Pakistan in collaborations with Dow University of Health Sciences Karachi, Pakistan. YT was funded by “Studienstiftung des deutschen Volkes.” YA was jointly funded by the Higher Education Ministry of Damascus University, Syria and the German Academic Exchange Service (DAAD). This work was supported by the University of Tuebingen “Projektförderung für NachwuchswissenschaftlerInnen 2012–2013.” SK is member of the DFG Research Training Group GRK1708. MHI was funded by the grant from the SYSTERACT project (ERASysAPP).

### Conflict of interest statement

The authors declare that the research was conducted in the absence of any commercial or financial relationships that could be construed as a potential conflict of interest.
