# Supplementary material for: Post-translational Serine/Threonine Phosphorylation and Lysine Acetylation: A Novel Regulatory Aspect of the Global Nitrogen Response Regulator GlnR in S. coelicolor M145
Source: Front Mol Biosci. 2016 Aug 9;3:38. doi: 10.3389/fmolb.2016.00038 (PMC4977719; doi:10.3389/fmolb.2016.00038)
Supplement: Supplementary file 5 [file Image1.PDF]

Post-translational lysine acetylation and serine/threonine phosphorylation modulate activity of GlnR in *S. coelicolor* M145

Rafat Amin [1], Mirita Franz-Wachtel [2],Yvonne Tiffert [3], Martin Heberer[1], Mohamed Meky[1], Yousra Ahmed [1,4], Arne Matthews[1], Sergii Krysenko[1], Marco Jakobi[1], Markus Hinder [1], Jane Moore [5], Nicole Okoniewski[1], Boris Maček [2], Wolfgang Wohlleben [1] and Agnieszka Bera [1*]

Supplementary Data

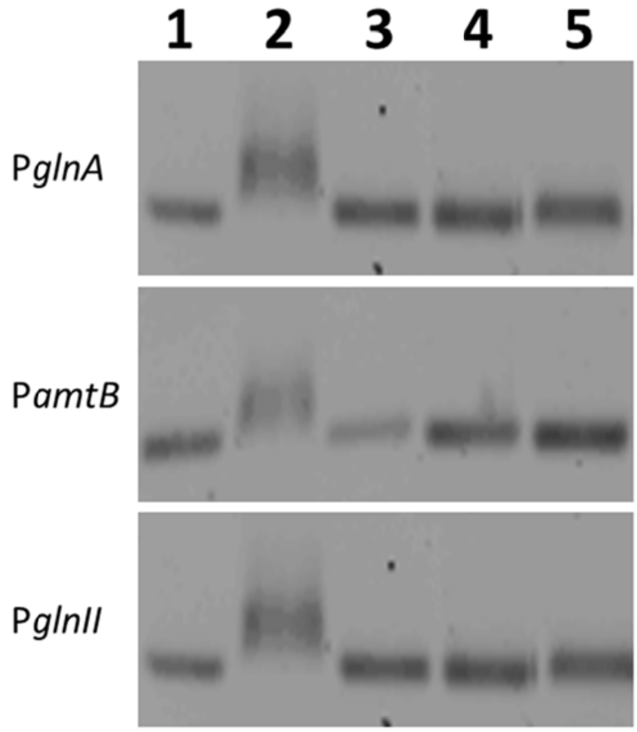

Fig. S1 **EMSAs with cell lysates generated from the *glnR* mutant.** Lane (1), control, promoter regions of the known GlnR target genes (Cy5 - labeled probe without cell lysate). Lane (2), Cy5 - labeled probe with cell lysate from the Strep-GlnR overexpression strain grown in Evans medium supplemented in 5 mM $NaNO_3$ – positive control. Lane (3-5), Cy5 - labeled probes with cell lysates from the *glnR* mutant (50µg of total proteins) grown in complex S-medium, Lane (3), the cell lysate (50µg of total proteins) from the *glnR* mutant grown in defined Evans medium with 5 mM $NaNO_{3,}$ Lane (4), the cell lysate (50µg of total proteins) from the *glnR* mutant grown in defined Evans medium with 100 mM $NaNO_{3,}$ Lane (5). EMSAs were performed in the presence of the 300X excess of the unlabeled, unspecific salmon sperm DNA.

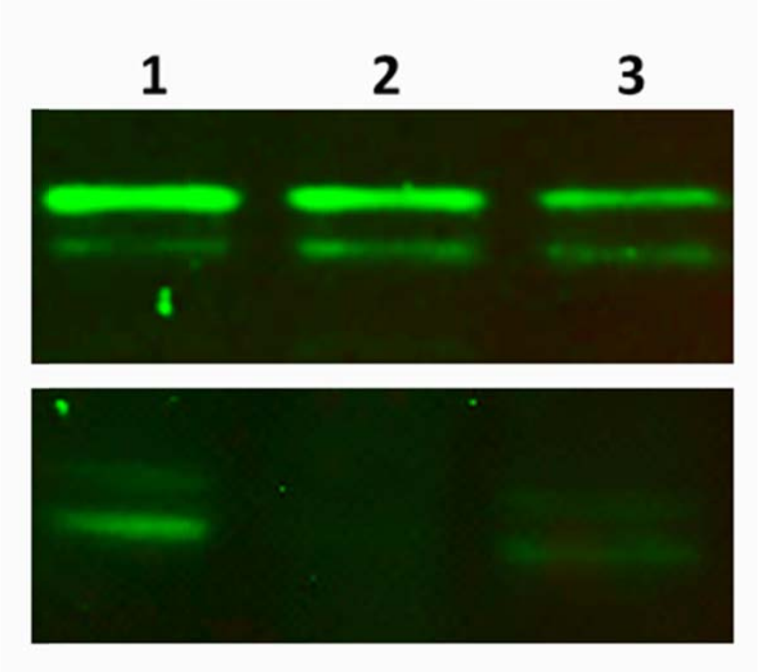

Fig. S2 ***In vitro* deacetylation of the GlnR.** 200-microliter reaction mixtures containing 100 µg GlnR, and 20µg CobB1 or CobB2 were incubated in the 50 mM Tris-HCl buffer pH 8.3 supplemented with 1 mM NAD^+. The deacetylation reaction was performed at 30°C for 6 h. The reactions were quenched with 50 µl of 5× SDS-PAGE loading buffer, and the mixtures were heated at 95°C for 5 min. Ten microliters were resolved by 12% SDS-PAGE, GlnR was detected by Western blot (top) using anti-GlnR antibodies generated in rabbit (1:5000) and the secondary goat anti-rabbit antibodies conjugated with HRP (1:3000), whereas acetylation was detected by Western blot (bottom) using anti-acetyl-lysine antibodies generated in rabbit (1:1000) and the secondary goat anti-rabbit antibodies conjugated with HRP (1:3000). Lane (1) Strep-GlnR incubated in the 50 mM Tris-HCl buffer pH 8.3 supplemented with 1 mM NAD^+, Lane (2) Strep-GlnR + CobB2 incubated in the 50 mM Tris-HCl buffer pH 8.3 supplemented with 1 mM NAD^+, Lane (3) Strep-GlnR + CobB1 incubated in the 50 mM Tris-HCl buffer pH 8.3 supplemented with 1 mM NAD^+.
