# Supplementary material for: Post-translational Serine/Threonine Phosphorylation and Lysine Acetylation: A Novel Regulatory Aspect of the Global Nitrogen Response Regulator GlnR in S. coelicolor M145
Source: Front Mol Biosci. 2016 Aug 9;3:38. doi: 10.3389/fmolb.2016.00038 (PMC4977719; doi:10.3389/fmolb.2016.00038)
Supplement: Supplementary file 6 [file Image2.PDF]

```
sma_SAV_4042          MSSLLLLTNALQPSTEVLPALGLLLHNVRVAPAEGPALVDTPGADVILIDGR
scb_SCAB_49561        MSSLLLLTNALQPSTEVLPALGLLLHNVRVAPAEGPALVDTPGADVILIDGR
sho_SHJGH_4686        MSSLLLLTNALQPSTEVLPALGLLLHNVRVAPAEGPALVDTPGADVILVDGR
shy_SHJG_4923         MSSLLLLTNALQPSTEVLPALGLLLHNVRVAPAEGPALVDTPGADVILVDGR
srw_TUE45_04698       MSSLLLLTNALQPSTEVLPALGLLLHNVRVAPAEGPALVDTPGADVILVDGR
sci_B446_18010        MSSLLLLTNALQPSTEVLPALGLLLHNVRVAPAEGPALVDTPGADVILIDGR
sdv_BN159_4795        MSSLLLLTNALQPSTEVLPALGLLLHNVRVAPAEGPALVDTPGADVILIDGR
scx_AS200_24775       MSSLLLLTNALQPSTEVLPALGLLLHNVRVAPAEGPALVDTPGADVILIDGR
strf_ASR50_18055      MSSLLLLTNALQPSTEVLPALGLLLHNVRVAPAEGPALVDTPGADVILIDGR
sgb_WQO_15815         MSSLLLLTNALQPSTEVLPALGLLLHSVRVAPAEGPALVDTPGADVILVDGR
sgr_SGR_3960          MSSLLLLTNALQPSTEVLPALGLLLHSVRVAPAEGPALVDTPGADVILVDGR
scz_ABE83_16450       MSSLLLLTNALQPSTEVLPALGLLLHSVRVAPAEGPALVDTPGADVILIDGR
sfi_SFUL_3958         MSSLLLLTNALQPSTEVLPALGLLLHSVRVAPAEGPALVDTPGADVILIDGR
strp_F750_3905        MSSLLLLTNALQPSTEVLPALGLLLHSVRVAPAEGPALVDTPGADVILIDGR
sfa_Sfla_2887         MSSLLLLTNALQPSTEVLPALGLLLHSVRVAPAEGPALVDTPGADVILIDGR
ssx_SACTE_3596        MSSLLLLTNALQPSTEVLPALGLLLHSVRVAPAEGPALVDTPGADVILIDGR
strm_M444_16555       MSSLLLLTNALQPSTEVLPALGLLLHNVRVAPAEGPALVDTPGADVILIDGR
spri_SPRI_4013        MSSLLLLTNALQPSTEVLPALGLLLHNVRVAPAEGPALVDTPGADVILVDGR
svt_SVTN_20470        MSSLLLLTNALQPSTEVLPALGLLLHNVRVAPAEGPALVDTPGADVILVDGR
sve_SVEN_3917         MSALLLLTNALQPSTEVLPALGLLLHNVRVAPAEGPALVDTPGADVILVDGR
sgu_SGLAU_15880       MSSLLLLTNALQPSTEVLPALGLLLHNVRVAPAEGPALVDTPGADVILIDGR
salb_XNR_3323         MSSLLLLTNALQPSTEVLPALGLLLHQVRVAPAEGPALVDTPGADVILIDGR
sbh_SBI_05051         MSSLLLLTNALQPSTEVLPALGLLLHGVRVAPAEGPALVDTPGADVILIDGR
scy_SCATT_25430       MSSLLLLTNALQPSTEVLPALGLLLHGVRVAPAEGPALVDTPGADVILVDGR
sct_SCAT_2553         MSSLLLLTNALQPSTEVLPALGLLLHGVRVAPAEGPALVDTPGADVILVDGR
strc_AA958_13605      MSSLLLLTNALQPSAEVLPALGLLLHNVRVAPAEGPALIDTPGADVILVDGR
sco_SCO4159           MSSLLLLTNALQPSTEVLPALGLLLHNVRVAPAEGPALVDTPGADVILVDGR
slv_SLIV_17580        MSSLLLLTNALQPSTEVLPALGLLLHNVRVAPAEGPALVDTPGADVILVDGR
samb_SAM23877_3522    MSSLLLLTNALQPSTEVLPALGLLLHNVRVAPAEGPALVDTPGADVILVDGR
scw_TU94_17830        MSSLLLLTNALQPSTEVLPALGLLLHHVRVAPAEGPALVDTPGADVILVDGR
sals_SLNWT_4064       MSSLLLLTNALQPSTEVLPALGLLLHNVRVAPAEGPALVDTPGADVILIDGR
sxi_SXIM_30940        MSSLLLLTNALQPSSQVLPALELLLHNVRVAPAEGPALVDAPSADVILIDGR
src_M271_22760        MSSLLLLTNALQPSTEVLPALGLLLHSVRVAPAEGPALVDTPGADVILIDGR
svl_Strvi_0642        MSSLLLLTNALQPSTEVLPALGLLLHSVRVAPAEGPALVDTPGADVILIDGR
sall_SAZ_25160        MSSLLLLTNALQPSTEVLPALGLLLHSVRVAPAEGPALIDTPGADVILIDGR
salu_DC74_4717        MSSLLLLTNALQPSTEVLPALGLLLHSVRVAPAEGPALIDTPGADVILIDGR
stre_GZL_04135        MSSLLLLTNALQPSTEVLPALGLLLHSVRVAPAEGPALIDTPGADVILIDGR
sld_T261_3541         MSSLLLLTNALQPSTEVLPALGLLLHSVRVAPAEGPALIDTPGADVILIDGR

sma_SAV_4042          LCQLLRSTGPGCPLILVVTEGGLAAVTADWGIDDVLLDTAGPAEVEARLRLA
scb_SCAB_49561        LCQLLRSTGPGCPLILVVTEGGLAAVTADWGIDDVLLDTAGPAEVEARLRLA
sho_SHJGH_4686        LCQLLRSTGPGCPLLLVVTEGGLAAVTADWGIDDVLLDTAGPAEVEARLRLA
shy_SHJG_4923         LCQLLRSTGPGCPLLLVVTEGGLAAVTADWGIDDVLLDTAGPAEVEARLRLA
srw_TUE45_04698       LCQLLRSTGPGCPLLLVVTEGGLAAVTADWGIDDVLLDTAGPAEVEARLRLA
sci_B446_18010        LCQLLRSTGPGCPLLLVVTEGGLAAVTADWGIDDVLLDTAGPAEVEARLRLA
sdv_BN159_4795        LCQLLRSTGPGCPLILVVTEGGLAAVTADWGIDDVLLDTAGPAEVEARLRLA
scx_AS200_24775       LCQLLRSTGPGCPLILVVTEGGLAAVTADWGIDDVLLDTAGPAEVEARLRLA
strf_ASR50_18055      LCQLLRSTGPGCPLVLVVTEGGLAAVTADWGIDDVLLDTAGPAEVEARLRLA
sgb_WQO_15815         LCQLLRSTGPGCPLILVVTEGGLAAVTADWGVDDVLLDTAGPAEVEARLRLA
sgr_SGR_3960          LCQLLRSTGLGCPLILVVTEGGLAAVTADWGVDDVLLDTAGPAEVEARLRLA
scz_ABE83_16450       LCQLLRSTGPGCPLILVVTEGGLAAVTADWGIDDVLLDTAGPAEVEARLRLA
sfi_SFUL_3958         LCQLLRSTGPGCPLILVVTEGGLAAVTADWGIDDVLLDTAGPAEVEARLRLA
strp_F750_3905        LCQLLRSTGPGCPLILVVTEGGLAAVTADWGIDDVLLDTAGPAEVEARLRLA
sfa_Sfla_2887         LCQLLRSTGPGCPLILVVTEGGLAAVTADWGIDDVLLDTAGPAEVEARLRLA
ssx_SACTE_3596        LCQLLRSTGPGCPLILVVTEGGLAAVTADWGIDDVLLDTAGPAEVEARLRLA
strm_M444_16555       LCQLLRSTGPGCPLILVVTEGGLAAVTADWGIDDVLLDTAGPAEVEARLRLA
spri_SPRI_4013        LCQLLRSTGPGCPLVLVVTEGGLAAVTADWGIDDVLLDTAGPAEVEARLRLA
svt_SVTN_20470        LCQLLRSTGPGCPLILVVTEGGLAAVTADWGIDDVLLDTAGPAEVEARLRLA
sve_SVEN_3917         LCQLLRSTGPGCPLILVVTEGGLAAVTADWGIDDVLLDTAGPAEVEARLRLA
sgu_SGLAU_15880       LCQLLRSTGPGCPLVLVVTEGGLAAVTADWGIDDVLLDTAGPAEVEARLRLA
```

```
salb_XNR_3323          LCQLLRSTGPGCPLVLVVTEGGLAAVTADWGIDDVLLDTAGPAEVEARLRLA
sbh_SBI_05051          LCQLLRSTGPGCPLILVVTEGGLAAVTADWGIDDVLLDTAGPAEVEARLRLA
scy_SCATT_25430        LCQLLRSTGPGCPLILVVTEGGLAAVTADWGVDDVLLDTAGPAEVEARLRLA
sct_SCAT_2553          LCQLLRSTGPGCPLILVVTEGGLAAVTADWGVDDVLLDTAGPAEVEARLRLA
strc_AA958_13605       LCQLLRSTGPGCPLILVVTEGGLAAVTAEWGVDDVLLDTAGPAEVEARLRLA
sco_SCO4159            LCQLLRSTGLSCPLVLIVTEGGLAAVTADWGIDDVLLDTAGPAEVEARLRLA
slv_SLIV_17580         LCQLLRSTGLSCPLVLIVTEGGLAAVTADWGIDDVLLDTAGPAEVEARLRLA
samb_SAM23877_3522     LCQLLRSTGLSCPLVLVVTEGGLAAVTADWGIDDVLLDTAGPAEVEARLRLA
scw_TU94_17830         LCQLLRSTGPGCPLLLVVTEGGLAAVTADWGIDDVLLDTAGPAEVEARLRLA
sals_SLNWT_4064        LCQLLRSTGPGCPLVLVVTEGGLAAVTADWGIDDVLLDTAGPAEVEARLRLA
sxi_SXIM_30940         LCQLLRSTGPGCPLLLIVTEGGLAAVTADWGVDDVVLDTAGPAEVEARLRLA
src_M271_22760         LCQLLRSTGPGCPLILVVTEGGLAAVTADWGIDDVLLDTAGPAEVEARLRLA
svl_Strvi_0642         LCQLLRSTGPGCPLILVVTEGGLAAVTADWGVDDVLLDTAGPAEVEARLRLA
sall_SAZ_25160         LCQLLRSTGPGCPLVLVVTEGGLAAVTADWGVDDVLLDTAGPAEVEARLRLA
salu_DC74_4717         LCQLLRSTGPGCPLVLVVTEGGLAAVTADWGVDDVLLDTAGPAEVEARLRLA
stre_GZL_04135         LCQLLRSTGPGCPLVLVVTEGGLAAVTADWGVDDVMLDTAGPAEVEARLRLA
sld_T261_3541          LCQLLRSTGPGCPLVLVVTEGGLAAVTADWGIDDVLLDTAGPAEVEARLRLA

                                   133  138 140 142     153   159
sma_SAV_4042           DDSPMEIRNGDLSVDEATYSAKLKGRVLDLTFKEFELLKYLAQHPGRVFTRA
scb_SCAB_49561         DDSPMEIRNGDLSVDEATYSAKLKGRVLDLTFKEFELLKYLAQHPGRVFTRA
sho_SHJGH_4686         DDSPMEIRNGDLSVDEATYSAKLKGRVLDLTFKEFELLKYLAQHPGRVFTRA
shy_SHJG_4923          DDSPMEIRNGDLSVDEATYSAKLKGRVLDLTFKEFELLKYLAQHPGRVFTRA
srw_TUE45_04698        DDSPMEIRNGDLSVDEATYSAKLKGRVLDLTFKEFELLKYLAQHPGRVFTRA
sci_B446_18010         DDSPMEIRNGDLSVDEATYSAKLKGRVLDLTFKEFELLKYLAQHPGRVFTRA
sdv_BN159_4795         DDSPMEIRNGDLSVDEATYSAKLKGRVLDLTFKEFELLKYLAQHPGRVFTRA
scx_AS200_24775        DDSPMEIRNGDLSVDEATYSAKLKGRVLDLTFKEFELLKYLAQHPGRVFTRA
strf_ASR50_18055       DDSPMEIRNGDLSVDEATYSAKLKGRVLDLTFKEFELLKYLAQHPGRVFTRA
sgb_WQO_15815          DDSPMEIRNGDLSVDEATYSAKLKGRVLDLTFKEFELLKYLAQHPGRVFTRA
sgr_SGR_3960           DASPMEIRNGDLSVDEATYSAKLKGRVLDLTFKEFELLKYLAQHPGRVFTRA
scz_ABE83_16450        DDSPMEIRNGDLSVDEATYSAKLKGRVLDLTFKEFELLKYLAQHPGRVFTRA
sfi_SFUL_3958          DDSPMEIRNGDLSVDEATYSAKLKGRVLDLTFKEFELLKYLAQHPGRVFTRA
strp_F750_3905         DDSPMEIRNGDLSVDEATYSAKLKGRVLDLTFKEFELLKYLAQHPGRVFTRA
sfa_Sfla_2887          DDSPMEIRNGDLSVDEATYSAKLKGRVLDLTFKEFELLKYLAQHPGRVFTRA
ssx_SACTE_3596         DDSPMEIRNGDLSVDEATYSAKLKGRVLDLTFKEFELLKYLAQHPGRVFTRA
strm_M444_16555        DDSPMEIRNGDLSVDEATYSAKLKGRVLDLTFKEFELLKYLAQHPGRVFTRA
spri_SPRI_4013         DDSPMEIRNGDLSVDEATYSAKLKGRVLDLTFKEFELLKYLAQHPGRVFTRA
svt_SVTN_20470         DDSPMEIRNGDLSVDEATYSAKLKGRVLDLTFKEFELLKYLAQHPGRVFTRA
sve_SVEN_3917          DDSPMEIRNGDLSVDEATYSAKLKGRVLDLTFKEFELLKYLAQHPGRVFTRA
sgu_SGLAU_15880        DDSPMEIRNGDLSVDEATYSAKLKGRVLDLTFKEFELLKYLAQHPGRVFTRA
salb_XNR_3323          DDSPMEIRNGDLSVDEATYSAKLKGRILDLTFKEFELLKYLAQHPGRVFTRA
sbh_SBI_05051          DDSPMEIRNGDLSVDEATYSAKLKGRVLDLTFKEFELLKYLAQHPGRVFTRA
scy_SCATT_25430        DDSPSEIRNGDLSVDEATYSAKLKGRVLDLTFKEFELLKYLAQHPGRVFTRA
sct_SCAT_2553          DDSPSEIRNGDLSVDEATYSAKLKGRVLDLTFKEFELLKYLAQHPGRVFTRA
strc_AA958_13605       DESPTEIRNGDLTVDEATYSAKLKGRVLDLTFKEFELLKYLAQHPGRVFTRA
sco_SCO4159            DDSPMEIRNGDLSVDEATYSAKLKGRVLDLTFKEFELLKYLAQHPGRVFTRA
slv_SLIV_17580         DDSPMEIRNGDLSVDEATYSAKLKGRVLDLTFKEFELLKYLAQHPGRVFTRA
samb_SAM23877_3522     DDSPMEIRNGDLSVDEATYSAKLKGRVLDLTFKEFELLKYLAQHPGRVFTRA
scw_TU94_17830         DDSPMEIRNGDLSVDEATYSAKLKGRVLDLTFKEFELLKYLAQHPGRVFTRA
sals_SLNWT_4064        DDSPMEIRNGDLSVDEATYSAKLKGRVLDLTFKEFELLKYLAQHPGRVFTRA
sxi_SXIM_30940         DDSPMEIRNGDLSVDEATYSAKLKGRVLDLTFKEFELLKYLAQHPGRVFTRA
src_M271_22760         DDSPMEIRNGDLSVDEATYSAKLKGRVLDLTFKEFELLKYLAQHPGRVFTRA
svl_Strvi_0642         DDSPTEIRNGDLSVDEATYSAKLKGRVLDLTFKEFELLKYLAQHPGRVFTRA
sall_SAZ_25160         DNGPTEIRTGDLSVDEATYSAKLKGRVLDLTFKEFELLKYLAQHPGRVFTRA
salu_DC74_4717         DNGPTEIRTGDLSVDEATYSAKLKGRVLDLTFKEFELLKYLAQHPGRVFTRA
stre_GZL_04135         DNGPMEIRTGDLSVDEATYSAKLKGRVLDLTFKEFELLKYLAQHPGRVFTRA
sld_T261_3541          DDGPMEIRNGDLSVDEATYSAKLKGRILDLTFKEFELLKYLAQHPGRVFTRA

                                         200    207  211
sma_SAV_4042           YDYFGGTRTVDVHVRRLRAKLGPEHESLIGTVRNVGYRFVTPEKGERGGGS-
scb_SCAB_49561         YDYFGGTRTVDVHVRRLRAKLGPEHESLIGTVRNVGYRFVTPEKAERAG---
sho_SHJGH_4686         YDYFGGTRTVDVHVRRLRAKLGPEHESLIGTVRNVGYRFVTPEKPDRSA---
shy_SHJG_4923          YDYFGGTRTVDVHVRRLRAKLGPEHESLIGTVRNVGYRFVTPEKPDRSA---
```

```
                                                 200       207 211
srw_TUE45_04698        YDYFGGTRTVDVHVRRLRAKLGPEHESLIGTVRNVGYRFVTPEKTDRSA---
sci_B446_18010         YDYFGGTRTVDVHVRRLRAKLGPEHESLIGTVRNVGYRFVTPEKGDRSG---
sdv_BN159_4795         YDYFGGTRTVDVHVRRLRAKLGPEHESLIGTVRNVGYRFVTPEKGDRAA---
scx_AS200_24775        YDYFGGTRTVDVHVRRLRAKLGPEHESLIGTVRNVGYRFVTPEKADRVP---
strf_ASR50_18055       YDYFGGTRTVDVHVRRLRAKLGPEHESLIGTVRNVGYRFVTPEKVERAA---
sgb_WQO_15815          YDYFGGTRTVDVHVRRLRAKLGPEHESLIGTVRNVGYRFVVPEKVERAAEE-
sgr_SGR_3960           YDYFGGTRTVDVHVRRLRAKLGPEHESLIGTVRNVGYRFVTPEKVERAAEE-
scz_ABE83_16450        YDYFGGTRTVDVHVRRLRAKLGPEHESLIGTVRNVGYRFVVPEKVERAAEE-
sfi_SFUL_3958          YDYFGGTRTVDVHVRRLRAKLGPEHESLIGTVRNVGYRFVTPEKVERAAEE-
strp_F750_3905         YDYFGGTRTVDVHVRRLRAKLGPEHESLIGTVRNVGYRFVTPEKVDRAAEE-
sfa_Sfla_2887          YDYFGGTRTVDVHVRRLRAKLGPEHESLIGTVRNVGYRFVTPEKVDRAAEE-
ssx_SACTE_3596         YDYFGGTRTVDVHVRRLRAKLGPEHESLIGTVRNVGYRFVTPEKPERPTED-
strm_M444_16555        YDYFGGTRTVDVHVRRLRAKLGPEHESLIGTVRNVGYRFVTPEKVERAAAE-
spri_SPRI_4013         YDYFGGTRTVDVHVRRLRAKLGPEHESLIGTVRNVGYRFVAPEKVERAAEE-
svt_SVTN_20470         YDYFGGTRTVDVHVRRLRAKLGPEHESLIGTVRNVGYRFVTPEKVERAAEE-
sve_SVEN_3917          YDYFGGTRTVDVHVRRLRAKLGPEHESLIGTVRNVGYRFVTPEKVERAAEE-
sgu_SGLAU_15880        YDYFGGTRTVDVHVRRLRAKLGPEHESLIGTVRNVGYRFVTPEKVERAAEE-
salb_XNR_3323          YDYFGGTRTVDVHVRRLRAKLGPEHESLIGTVRNVGYRFVTPEK-----PE-
sbh_SBI_05051          YDYFGGTRTVDVHVRRLRAKLGPEHESLIGTVRNVGYRFVSPEKGDRGGEE-
scy_SCATT_25430        YDYFGGTRTVDVHVRRLRAKLGPEHESLIGTVRNVGYRFVSPEKEKGEQRT-
sct_SCAT_2553          YDYFGGTRTVDVHVRRLRAKLGPEHESLIGTVRNVGYRFVSPEKEKGEQRT-
strc_AA958_13605       YDYFGGTRTVDVHVRRLRAKLGPEHESLIGTVRNVGYRFVVPE--KGEKKP-
sco_SCO4159            YDYFGGTRTVDVHVRRLRAKLGPEHESLIGTVRNVGYRFVTPEKPEKGEK--
slv_SLIV_17580         YDYFGGTRTVDVHVRRLRAKLGPEHESLIGTVRNVGYRFVTPEKPEKGEK--
samb_SAM23877_3522     YDYFGGTRTVDVHVRRLRAKLGPEHESLIGTVRNVGYRFVTPEKPEKPQK--
scw_TU94_17830         YDYFGGTRTVDVHVRRLRAKLGPEHESLIGTVRNVGYRFVTPEKPEKPAR--
sals_SLNWT_4064        YDYFGGTRTVDVHVRRLRAKLGPEHESLIGTVRNVGYRFVTPEKPDRGASA-
sxi_SXIM_30940         YDYFGGTRTVDVHVRRLRAKLGPEHESLIGTVRNVGYRFVVPEK--------
src_M271_22760         YDYFGGTRTVDVHVRRLRAKLGPEHESLIGTVRNVGYRFVAPEKVERAAEE-
svl_Strvi_0642         YDYFGGTRTVDVHVRRLRAKLGPEHESLIGTVRNVGYRFVAPEKVERAAEE-
sall_SAZ_25160         YDYFGGTRTVDVHVRRLRAKLGPEHESLIGTVRNVGYRFVVPEKVERAAAE-
salu_DC74_4717         YDYFGGTRTVDVHVRRLRAKLGPEHESLIGTVRNVGYRFVVPEKVERAAAE-
stre_GZL_04135         YDYFGGTRTVDVHVRRLRAKLGPEHESLIGTVRNVGYRFVVPEKVERAAAE-
sld_T261_3541          YDYFGGTRTVDVHVRRLRAKLGPEHESLIGTVRNVGYRFVTPEKAERAAEDR

sma_SAV_4042           GTSGPAA---IPAKPEDA--------DETAHVVASEVTAEA---------
scb_SCAB_49561         SS-----------KAADA--------DETAPLEAVEVAAEA---------
sho_SHJGH_4686         RA-----------KPEDA--------DAPAVLEGTEVPAEA---------
shy_SHJG_4923          RA-----------KPEDA--------DAPAVLEGTEVPAEA---------
srw_TUE45_04698        RA-----------KAEDA--------DAPAVLDGTEVHAEA---------
sci_B446_18010         RA-----------KADDA--------DTSAVLDGVELHADA---------
sdv_BN159_4795         RQ-----------KAADT--------DETAVLDGVEVSADA---------
scx_AS200_24775        RA-----------KAEDA--------DTASARDGAEVPADA---------
strf_ASR50_18055       RQ-----------KAAEA--------DETAAADADGVRAKA---------
sgb_WQO_15815          QKADGPEARPVARSEQSS--------SATAEEEAPVEAAKR---------
sgr_SGR_3960           QRAAG----PVARSEQSS--------SATATEEAPVEAAKR---------
scz_ABE83_16450        QKADD----PVARSEQSS--------SATAEEEAPVRAAKG---------
sfi_SFUL_3958          QKAAE----PVTRSEQSS--------SATAAEEAPVEAAKR---------
strp_F750_3905         PTDDL-----VTRSEQSA--------DIEVTEEAPVRPAKR---------
sfa_Sfla_2887          PTDDL-----VTRSEQSA--------DTEVPEEAPVRPAKR---------
ssx_SACTE_3596         AAGET-----ATRSEQSD--------QARISEEAPVRPAKR---------
strm_M444_16555        AAAQSAR-QASASVTRTE--------ESPAIVPSGGRPAQR---------
spri_SPRI_4013         ------------AVARTD--------DGAEVEEPAVRPAQR---------
svt_SVTN_20470         RAKEE-----ARVSARVK--------GDTEAVTPT---------------
sve_SVEN_3917          RVKEG-----ARANAGVK--------DATGDVTPAEDRNVADATVRPAGR
sgu_SGLAU_15880        RDNTN-----KTDKADSL--------SASVATDA----------------
salb_XNR_3323          ----AKEQAQEEAAEKVR--------ESRAASGSTVTEKAAAKP------
sbh_SBI_05051          ----GTGRAAPEAAPADG--------APRAENMAENMKAGAARPANT---
scy_SCATT_25430        GAQPAEARTGASRTAGAR--------AGSARTGGSTTGEGAGQPA-----
sct_SCAT_2553          GAQPAEARTGASRTAGAR--------AGSARTGGSTTGEGAGQPA-----
strc_AA958_13605       ----KQEQKGAAQEARHR--------A-----------------------
```

```
                                                         256        264/5
sco_SCO4159           SDKSEKAERAEKAERAET-------PGKAAAETNEAAGARSSKV-----
slv_SLIV_17580        SDKSEKGERAEKAERAET-------PGKAAAETNEAAGARPSKV-----
samb_SAM23877_3522    TER--------AAEDAKA-------EARPAAQADTTAGAASTKV-----
scw_TU94_17830        AAEEAASPATGRAKRGEA-------DTAAAAAVAEVPAEA---------
sals_SLNWT_4064       AGKSAKSGAKQDDSDEDA-------AARVVAEAEAAVRPAKR-------
sxi_SXIM_30940        -----------EKPADDH-------ARAVTREAEQLVKEAARRA-----
src_M271_22760        DAGAGGAGRGGPPDGAAGSAGSAGSAGSAARTAAARPANS---------
svl_Strvi_0642        GAAPGDAARGEHPGEAP---------GSVARAAAARPANS---------
sall_SAZ_25160        KAAK------DGARAKRA-------DGRAPARAASTGAGSAPAQPAGR-
salu_DC74_4717        KAAK------DGARAKRA-------DGRAPARAASTGAGSAPAQPAGR-
stre_GZL_04135        KATKAAKAVGDGDGGTRA-------DGPTSVRSPSSGAGSTSAQPARR-
sld_T261_3541         KAAS-------DDRGTPR-------DGGGSGKAVAARAESAARGPASR-
```

Fig. S3 Alignment of the GlnR homologous protein sequences from 37 Streptomyces sp. Alignment of the GlnR-protein sequences (http://www.genome.jp/kegg/) was performed using Clustal W and Boxshade (http://mobyle.pasteur.fr). Conserved residues are shown as white letter on black background. Non-conserved residues are shown as black letters on a white background. Black letters on gray background indicate similar amino acids. Phosphorylated serine/threonine residues are highlighted in red. Acetylated lysine residues are highlighted in green. GlnR from S. coelicolor is highlighted in green (sco_SCO4159).
