# Supplementary material for: Post-translational Serine/Threonine Phosphorylation and Lysine Acetylation: A Novel Regulatory Aspect of the Global Nitrogen Response Regulator GlnR in S. coelicolor M145
Source: Front Mol Biosci. 2016 Aug 9;3:38. doi: 10.3389/fmolb.2016.00038 (PMC4977719; doi:10.3389/fmolb.2016.00038)
Supplement: Supplementary file 7 [file Image3.PDF]

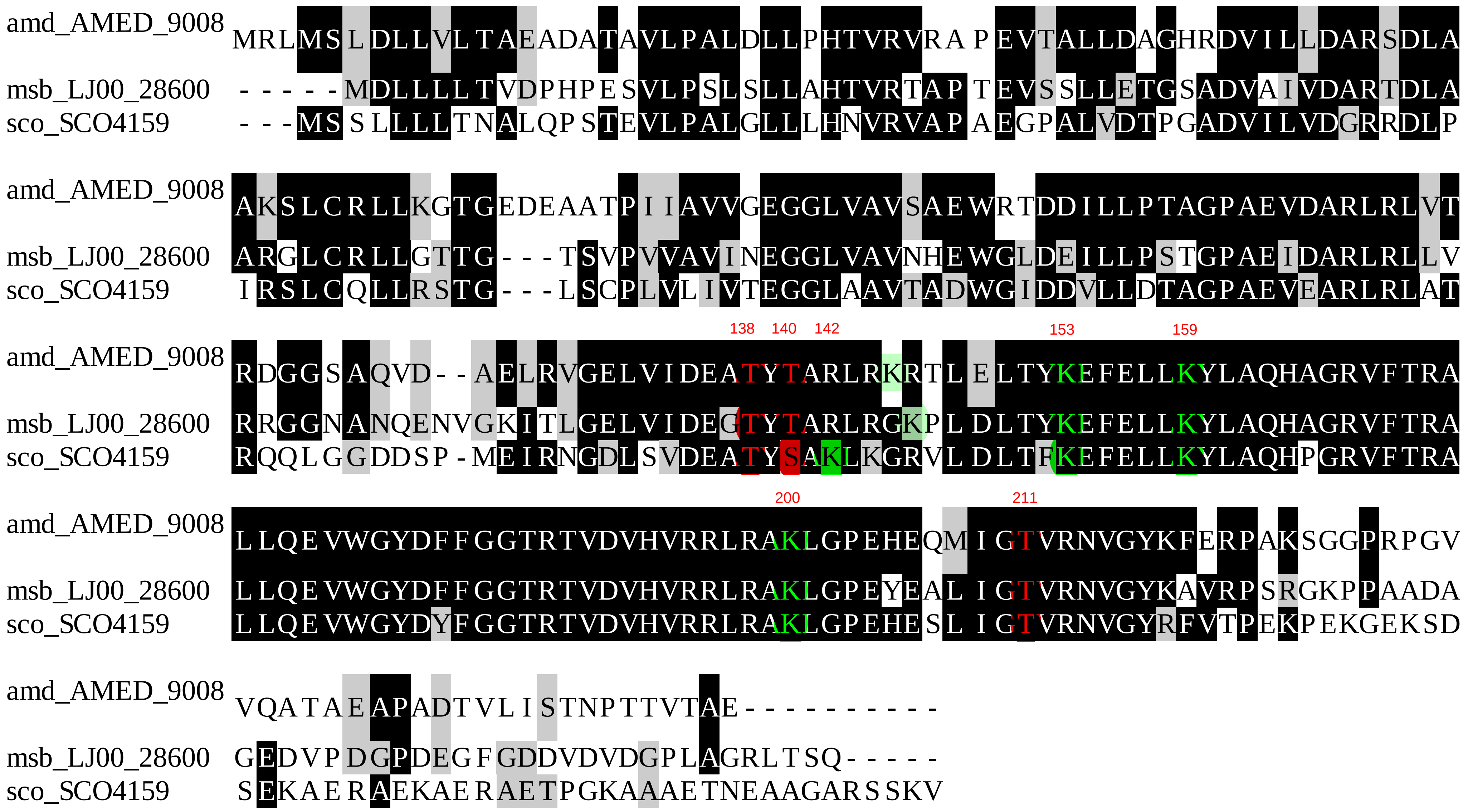

Fig. S4 Alignment of the GlnR homologous protein sequences from S. coelicolor (SCO4159), M. smegmatis (LJ00_28600) and A. mediterranei U32 (AMED_9008). Alignment of the GlnR-protein sequences (http://www.genome.jp/kegg/) was performed using Clustal W and Boxshade (http://mobyle.pasteur.fr). Conserved residues are shown as white letter on black background. Non-conserved residues are shown as black letters on a white background. Black letters on gray background indicate similar amino acids.Phosphorylated serine/threonine residues are highlighted in red. Acetylated lysine residues are highlighted in green. Positions of the modified residues are refered to GlnR-protein sequence from S.coelicolor.
